# Carbon Monoxide Promotes the Catalytic Hydrogenation on Metal Cluster Catalysts

**DOI:** 10.34133/2020/4172794

**Published:** 2020-07-17

**Authors:** Ruixuan Qin, Pei Wang, Pengxin Liu, Shiguang Mo, Yue Gong, Liting Ren, Chaofa Xu, Kunlong Liu, Lin Gu, Gang Fu, Nanfeng Zheng

**Affiliations:** ^1^State Key Laboratory for Physical Chemistry of Solid Surfaces, Collaborative Innovation Center of Chemistry for Energy Materials, and National & Local Joint Engineering Research Center for Preparation Technology of Nanomaterials, College of Chemistry and Chemical Engineering, Xiamen University, Xiamen 361005, China; ^2^College of Science, Huazhong Agricultural University, Wuhan 430070, China; ^3^Institute of Physics, Chinese Academy of Sciences, Beijing 100190, China

## Abstract

Size effect plays a crucial role in catalytic hydrogenation. The highly dispersed ultrasmall clusters with a limited number of metal atoms are one candidate of the next generation catalysts that bridge the single-atom metal catalysts and metal nanoparticles. However, for the unfavorable electronic property and their interaction with the substrates, they usually exhibit sluggish activity. Taking advantage of the small size, their catalytic property would be mediated by surface binding species. The combination of metal cluster coordination chemistry brings new opportunity. CO poisoning is notorious for Pt group metal catalysts as the strong adsorption of CO would block the active centers. In this work, we will demonstrate that CO could serve as a promoter for the catalytic hydrogenation when ultrasmall Pd clusters are employed. By means of DFT calculations, we show that Pd_*n*_ (*n* = 2‐147) clusters display sluggish activity for hydrogenation due to the too strong binding of hydrogen atom and reaction intermediates thereon, whereas introducing CO would reduce the binding energies of vicinal sites, thus enhancing the hydrogenation reaction. Experimentally, supported Pd_2_CO catalysts are fabricated by depositing preestablished [Pd_2_(*μ*-CO)_2_Cl_4_]^2-^ clusters on oxides and demonstrated as an outstanding catalyst for the hydrogenation of styrene. The promoting effect of CO is further verified experimentally by removing and reintroducing a proper amount of CO on the Pd cluster catalysts.

## 1. Introduction

Metal catalysts are widely used in industrial applications. Metal nanoparticles, clusters, and even atomically dispersed metal catalysts have been extensively explored for their high mass-specific activity [1–5]. For a wide range of reactions on metal surfaces, the adsorption energies and activation barriers are typically related to the Brønsted-Evans-Polanyi relationships [6–8]. As for metal catalysts with different sizes, their coordinative and electronic properties are often different from each other. For example, small metal clusters with a large part of coordinative unsaturated sites usually have stronger interaction energy with molecules than that of their larger counterparts [9, 10]. According to the Sabatier principle [11], the optimum catalytic performance can be achieved with a medium interaction energy such that volcano-shaped size-performance relationships would be observed in many heterogeneous catalytic reactions [12–16].

In addition to the size effect, the adsorbate-catalyst interaction also causes the electron redistribution in the unity. Similar to the ligands on homogenous catalysts, the coadsorbates on heterogeneous catalysts are able to modulate the electronic and coordinative structures as well, thus altering the adsorption energies of substrates and intermediates [17–21]. These effects will be more profound on ultrasmall clusters where the large ratio of surface atoms makes them an ingenious platform for modulating their electronic properties and thus catalytic performance through the surface coordination chemistry [22, 23].

In this work, through systematic density functional theory (DFT) calculations, we revealed that supported ultrasmall Pd clusters interacted too strongly with H atoms as well as reaction intermediates such that the hydrogenation activity was inhibited. When electron-withdrawing molecules (e.g., CO) were introduced, the adsorption energies of H atoms and hydrogenated intermediates on Pd clusters were weakened, thus boosting hydrogenation performance. Based on the theoretical analysis, oxide-supported Pd_2_ catalysts with CO binding were synthesized by using presynthesized carbonyl dipalladium clusters, [Pd_2_(*μ*-CO)_2_Cl_4_]^2-^, as the precursor. Indeed, the as-prepared Pd_2_CO catalyst exhibits dramatically enhanced hydrogenation activity. Further experiments revealed that a proper amount of preadsorbed CO showed positive effect for hydrogenation on metal cluster catalysts but negative effect on single-atom catalysts and negligible effect on nanocatalysts. We expected that the metal cluster catalysts with molecular modifiers would have more room to be tuned and bridge the gap of single-atom catalysts and nanosized catalysts.

## 2. Results

**Figure 1:**
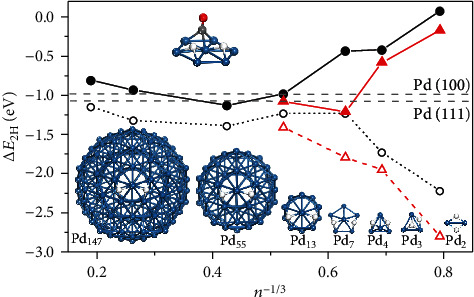
Adsorption energy of H_2_ on Pd with different sizes. Calculated Δ*E*_2H_ on bare Pd_*n*_ (marked in black circle) and TiO_2_(010)-supported Pd_*n*_ (marked in red triangle) clusters. The hollow and solid symbols are denoted as the CO-free and CO-modified cases, respectively. The gray dashed lines represent Δ*E*_2H_ on Pd(111) and Pd(100). The blue, dark gray, red, and white balls represent Pd, C, O, and H atoms. The size of Pd clusters was estimated by *n*^−1/3^.

It is widely accepted that the hydrogenation of C=C bonds follows the so-called Horiuti-Polanyi (H-P) mechanism, which consists of the successive addition of atomic hydrogen to the substrate. In this case, adsorption energies of H atoms on a metal surface turned out to be a critical descriptor [24–26]. In earlier works, the dissociation adsorption energy (Δ*E*_2H_) of H_2_ on Pd(111) and Pd(100) was calculated to be -1.08 eV and -0.98 eV, respectively [27–29]. Both the experiment and theoretical calculations showed that hydrogenation of alkenes would occur smoothly on both Pd(111) and Pd(100) [30]. In addition, it has also been reported that Δ*E*_2H_ decreased with the size decrease of Pd nanoparticles [31–33]. In order to calculate the dissociation adsorption energy of H_2_ on different Pd clusters, here, we constructed a set of Pd_*n*_ with size varying from Pd_2_ to Pd_147_. For simplicity, H atoms were placed on the most favorable sites in neighbor configuration.  Figure 1 plots the calculated Δ*E*_2H_ versus the reciprocal of the size of Pd_*n*_ clusters, i.e., *n*^−1/3^. According to our DFT calculations, despite the tortuous trends, Δ*E*_2H_ became much lower than that of Pd(111) and Pd(100) following the decreasing size of Pd clusters. To the extreme cases, Δ*E*_2H_ for Pd_2_ and Pd_3_ clusters were predicted to be as low as -2.22 eV and -1.73 eV, respectively. Such strong binding of H atoms indicated that ultrasmall Pd_*n*_ clusters would have poor activity towards hydrogenation.

**Figure 2:**
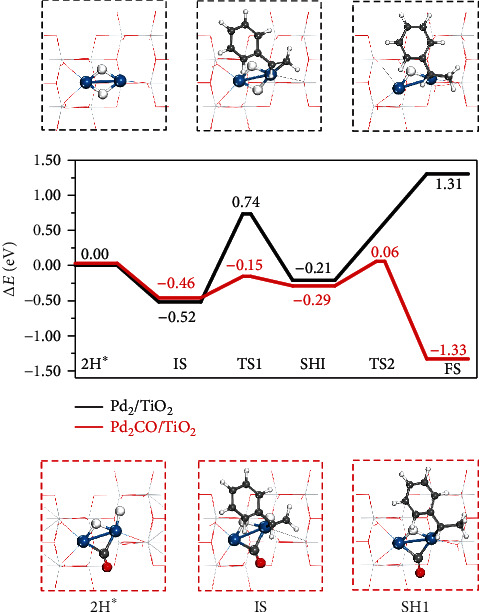
Theoretical calculation of styrene hydrogenation over Pd_2_ and Pd_2_CO. Energy profiles for styrene hydrogenation on Pd_2_/TiO_2_(010) (black) and Pd_2_CO/TiO_2_ (010) (red). IS denoted as the coadsorption of styrene and H atoms, SHI represented the semihydrogenated intermediate, and FS stood for the gaseous ethylbenzene with the H-free surface. The key structures were illustrated in the black and red dashed frame. The blue, red, dark gray, and white balls represented Pd, O, C, and H atoms, respectively.

For the practical using, Pd clusters should be loaded on supports such that the support effect on Δ*E*_2H_ should not be neglected [34–37]. Computationally, Pd_*n*_ (*n* = 2‐7) clusters were placed on the anatase TiO_2_(010) slab surface (Figures S1–S3). DFT calculations demonstrated that the geometries, the Pd-Pd distances, spin states, and the charge distribution of Pd_*n*_ clusters changed significantly as compared with their unsupported counterparts (Tables S2–S4). Unfortunately, upon being supported on oxide, Δ*E*_2H_ on the Pd clusters were decreased by 0.2~0.5 eV, further deviating from those of Pd(111) and Pd(100).  Figure 2 illustrates the calculated energy profile of catalytic hydrogenation of styrene over TiO_2_-supported Pd_2_ clusters (denoted as Pd_2_/TiO_2_). In the profile, Pd_2_ with the dissociated H atoms was used as the energy reference [27]. Styrene was then adsorbed with an adsorption energy of -0.52 eV, close to that on the H-covered Pd(100) surface (-0.43 eV). However, Pd_2_ and Pd(100) have dramatically different behaviors in the following hydrogenation steps. As illustrated in Figure 2, the first step hydrogenation (TS1) on Pd_2_/TiO_2_ involved a barrier of 1.26 eV (Figure S4). More severely, the second step was highly endothermic (1.52 eV), and no TS was able to be located despite our best efforts. These data indicated that not only H atom but also the alkyl radical was strongly bound on Pd_2_/TiO_2_ [38]. We also explored the hydrogenation of styrene on TiO_2_-supported Pd_3_ clusters (Pd_3_/TiO_2_) in which the two-step hydrogenation also exhibited high barriers of 0.70 eV (TS1) and 1.09 eV (TS2) (Figure S5 and S6). All these results suggested that the adsorption of H atoms and alkyl radical on small Pd_*n*_ clusters was too strong, far deviating from the volcano peak based on the Sabatier principle.

The disfavored binding of both H and alkyl radical is related to the electronic property of the Pd clusters [24, 38]. Thus, a possible way to remedy the hydrogenation activity of Pd_*n*_ clusters is to tune their electronic structures. It has been well documented that the adsorbed CO molecules can attract electrons from metal *d* orbitals [39, 40], thus regulating the adsorption energies of other coadsorbed species [17–21]. In this regard, introducing a proper amount of CO was expected to enhance the catalytic hydrogenation activity of Pd clusters. Computationally, one CO molecule was placed on the site nearby the H atom adsorption sites, sharing at least one Pd atom (Figures S7–S10). As shown in Figure 1, the presence of CO did increase Δ*E*_2H_ on Pd clusters significantly, especially for those ultrasmall Pd_*n*_ clusters (*n* = 2‐7). Similarly, the binding energy of styrene on the Pd clsuter was also reduced significantly by the cocoordination of CO (Figure S11). Inspired by this result, we revisited the catalytic hydrogenation on both Pd_2_/TiO_2_ and Pd_3_/TiO_2_ catalysts with Pd modified by CO, hereafter denoted as Pd_2_CO/TiO_2_ and Pd_3_CO/TiO_2_, respectively. The dissociation of H_2_ on Pd_2_CO/TiO_2_ and Pd_3_CO/TiO_2_ only needs to overcome small barriers of 0.35 eV and 0.18 eV (Figure S10), respectively, indicating that the H-P mechanism would still work on the CO-modified catalysts. As shown in Figure 2, on Pd_2_CO/TiO_2_, the reaction was downhill by 1.33 eV when going from the H atom adsorption state to the final state (FS), the production of ethylbenzene. As the result, the stepwise hydrogenation on Pd_2_CO/TiO_2_ exhibited very small barriers, 0.31 eV (TS1) and 0.35 eV (TS2) (Figure S4). A similar situation was found in Pd_3_CO/TiO_2_ catalyzed styrene hydrogenation, which was also exothermic by 0.91 eV. The calculated barriers for TS1 and TS2 were 0.81 eV and 0.54 eV (Figures S5 and S6), respectively. These findings indicated that introducing a proper amount of CO on Pd_*n*_ clusters would enhance the catalytic hydrogenation.

**Figure 3:**
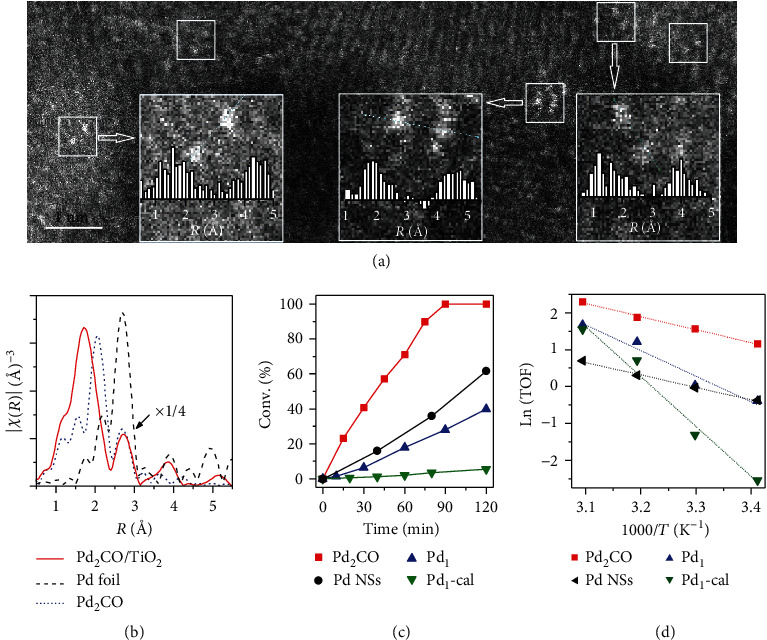
Structure characterization and styrene hydrogenation performance. (a) HAADF-STEM images of Pd_2_CO/TiO_2_. The three inserted images demonstrated the distances between two palladium atoms. (b) FT-EXAFS of Pd_2_CO/TiO_2_, palladium foil, and the Pd_2_CO, (PPh_4_)_2_[Pd_2_(*μ*-CO)_2_Cl_4_]. (c) Styrene conversion profiles and (d) corresponding Arrhenius plots of Pd_2_CO/TiO_2_ (Pd_2_CO), Pd nanosheets (Pd NSs), Pd_1_/TiO_2_-EG (Pd_1_), Pd_1_/TiO_2_-cal (Pd_1_-cal).

Inspired by the theoretical results, we synthesized Pd_2_CO/TiO_2_ by depositing a premade dipalladium complex, [Pd_2_(*μ*-CO)_2_Cl_4_]^2-^, on TiO_2_ [41, 42]. The THF solution of the metal precursor was added dropwise into the THF dispersion of TiO_2_ (Figures S12–S15) with *ca.* 0.2 wt% Pd loading. The scanning transmission electron microscope (STEM) energy dispersive X-ray (EDX) element mapping images in Figure S16 confirmed the highly dispersed Pd on TiO_2_, and no Pd nanoparticles were observed by HRTEM (Figure S17). As shown in Figure S18, the similar UV-vis spectrum of the as-obtained catalyst to that of the precursor in THF implied the mainly preserved coordination structure of the dinuclear Pd_2_ motifs. Indeed, high-angle annular dark-field (HAADF) STEM studies (Figure 3(a) and Figure S19) clearly revealed the dinuclear nature of Pd on TiO_2_, confirming that the main structure of the dinuclear Pd_2_ motifs was preserved upon deposition. The measured distance between the two nearby Pd atoms was about 2.7 Å, also consistent with that in the corresponding crystal structure (Figure S20 and Tables S5 and S6) and our calculations (2.72 Å, Table S2). As shown in the Fourier transform extended X-ray absorption fine structure (FT-EXAFS) in Figure 3(b), the Pd-Pd scattering shell was kept after loading. The fitted coordination number (CN) of Pd-Pd in the as-obtained Pd_2_CO/TiO_2_ was 1.2, close to that in the precursor (Figures S21 and S22, Table S7). We thus assumed that upon deposition, the [Pd_2_(*μ*-CO)_2_Cl_4_]^2-^ cluster reacted with the surface-adsorbed water or hydroxyl species with one of the CO ligands oxidized into CO_2_ (see Equation (1)), while the other CO remained on the deposited Pd cluster [43]. The retained CO was confirmed by DRIFTS and temperature programmed desorption-mass spectrometry (TPD-MS) (Figures S23–S25):

(1) $$\begin{array}{c} {\left[{\text{Pd}}_{2}{\left(\mu -\text{CO}\right)}_{2}{\text{Cl}}_{4}\right]}^{2-}+{\text{H}}_{2}\text{O}\to \text{P}{\text{d}}_{2}\left(\mu -\text{CO}\right)+\text{C}{\text{O}}_{2}+2{\text{H}}^{+}+4\text{C}{\text{l}}^{-} \end{array}$$

As shown in the diffuse reflectance infrared Fourier transform spectroscopy (DRIFTS) for Pd_2_CO/TiO_2_-catalyzed ethylene hydrogenation (Figure S26), the CO molecules inherited from the carbonyl precursors were nicely preserved on Pd. The catalytic activity of Pd_2_CO/TiO_2_ in the hydrogenation of styrene was evaluated and compared with those of single-atom Pd catalysts, such as Pd_1_/TiO_2_-EG (denoted as Pd_1_) with single-atom Pd on ethylene glycolate-stabilized TiO_2_(B) [43], and Pd_1_/TiO_2_-cal (denoted as Pd_1_-cal) with surface ethylene glycolate removed by calcination [44]. As shown in Figure 3(c), Pd_2_CO/TiO_2_ exhibited a much higher activity than the two single-atom Pd catalysts [45, 46]. The calculated TOFs based on the surface Pd atoms (Figure S27) revealed that, with the presence of CO binding, the supported Pd_2_ clusters were indeed as active as the surface Pd on large NPs. More importantly, the high Pd dispersion made the supported Pd_2_CO exhibit several times higher mass-specific activity (normalized by all Pd atoms in the catalyst). In addition, as shown in Figure 3(d), the apparent activation energy (*E*_a_) of Pd_2_CO/TiO_2_ (29.6 kJ/mol) was much smaller than the single-atom Pd catalysts (Pd_1_, 57.9 kJ/mol; Pd_1_-cal, 112.7 kJ/mol) and comparable to that of Pd nanosheets (28.2 kJ/mol). Furthermore, when D_2_ was used to replace H_2_ for the catalysis (Figure S28), a normal isotope effect of 2.02 was observed on Pd_2_CO/TiO_2_, confirming the homolytic activation mechanism of H_2_. Although the Pd-Pd coordination number was slightly changed (Figure S29, Table S7), Pd_2_CO/TiO_2_ maintained its high catalytic activity in 5 runs (Figure S30).

**Figure 4:**
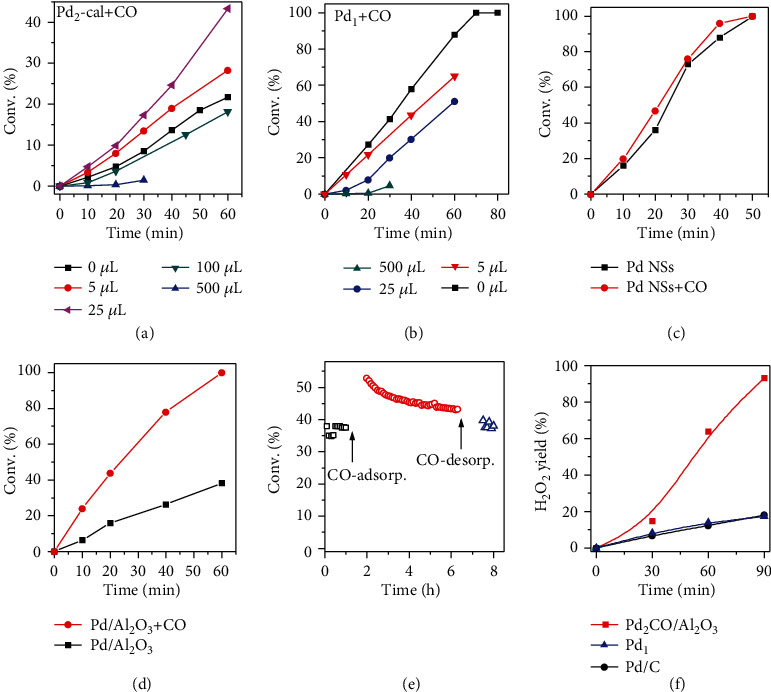
Effect of CO over different Pd catalysts. (a, b) Catalytic styrene hydrogenation performance of Pd_2_/TiO_2_-cal (*S*/*C* = 50,000) and Pd_1_/TiO_2_ (*S*/*C* = 10,000) with different amounts of CO introduced. (c, d) Catalytic styrene hydrogenation performance of Pd cubes and 0.5 wt% Pd/Al_2_O_3_ (*S*/*C* = 10,000) before and after treatment with CO (e) The steady state C_2_H_4_ hydrogenation test catalyzed by 0.5 wt% Pd/Al_2_O_3_ at 30°C. (f) H_2_O_2_ yield following the 2-eAQ hydrogenation route catalyzed by Pd_2_CO/Al_2_O_3_, Pd_1_/TiO_2_-EG, and Pd/C.

The promotional effect of CO on Pd clusters was further confirmed by removing CO from Pd_2_CO/TiO_2_ through calcination and readding an appropriate amount of CO to CO-removal catalysts. The presence of Pd-Pd scattering (CN≈4) in FT-EXAFS indicated the formation of larger Pd clusters (*ca.* a cluster with ~10-20 Pd atoms in average) after calcination and treatment with H_2_. After reintroducing CO, the bridge site and hollow site CO adsorbed on reduced Pd were also figured out in DRIFTS (Figure S31). Although no significant structure change of Pd clusters was observed in the FT-EXAFS, the slightly positive shift of XANES after introducing CO revealed the electronic transfer between the Pd clusters and CO (Figure S32 and Table S7). In styrene hydrogenation, the catalytic activity of Pd_2_/TiO_2_-cal was only ~1/5 of Pd_2_CO/TiO_2_ (Figure 4(a) and Figure S26). The apparent activation energy (80.1 kJ/mol, Figure S33) was much larger than that of Pd_2_CO/TiO_2_ and Pd nanosheets, implying that the bare Pd clusters were not efficient for hydrogenation, also echoing with our theoretical predictions. Interestingly, by introducing a proper amount of CO back to the CO-free system, the hydrogenation activity was enhanced by about twice (Figure 4(a)). In comparison, the activity of single-atom Pd catalysts was deterred significantly upon the introduction of CO, no matter for the calcined and uncalcined samples (Figure 4(b) and Figure S34a). It has been reported that the coordinated CO on the single-atom Ir could promote CO oxidation following the Eley-Rideal mechanism at the interfacial site [47], but the strongly coordinated CO on single-atom Pd will block the activation of H_2_ and inhibit the hydrogenation following the H-P mechanism. Interestingly, when Pd nanosheets and Pd nanocubes were used, the addition of CO would not retard much the hydrogenation (Figure 4(c) and Figure S34b). This can be explained by the possible formation of Pd hydride species upon the introduction of H_2_, which helps to weaken the CO binding on Pd and thus exhibits high CO tolerance [48]. Experimentally, the catalytic performance tests were conducted after long-time purging with H_2_, also helping to remove CO from the Pd surface.

More impressively, an Al_2_O_3_-supported Pd catalyst with 0.5 wt% mass loading and ~55% Pd dispersion was prepared (*ca.* ~1 nm, ~40 Pd atoms, Figures S35 and 36). The predominant H_2_ desorption temperature was decreased from about 175°C to 120°C with preadsorbed CO (Figure S37), verifying that the adsorption of CO reduced the binding energy of H over Pd clusters. Again, in styrene hydrogenation, 0.5 wt% Pd/Al_2_O_3_ displayed a three-time enhancement in the catalytic activity after being treated with CO (Figure 4(d)). Furthermore, as demonstrated in the *in situ* DRIFTS (Figure S38), the adsorbed bridge and hollow site CO on the reduced Pd surface was preserved on Pd/Al_2_O_3_ even after being heated up to 100°C in the condition of catalytic ethylene hydrogenation. Consequently, as shown in Figure 4(e), after the Pd/Al_2_O_3_ catalyst was treated with CO, the promotional effect kept for several hours, despite the gradual reduction of the promotional effect related to the desorption of partial CO during the exothermic ethylene hydrogenation. Once CO was removed, the activity decreased back to the initial value, excluding the probable structure change.

To expand the applications of the supported Pd_2_CO cluster, 0.2 wt% Pd_2_CO/Al_2_O_3_ was also fabricated using Al_2_O_3_ as the support (Figures S39 and S40 and Table S7). Pd_2_CO/Al_2_O_3_ exhibited almost the same catalytic activity as Pd_2_CO/TiO_2_ in styrene hydrogenation (Figure S41), indicating that it was the CO ligand but not the support promoting the catalysis of the Pd clusters. As demonstrated in the H_2_O_2_ production through the 2-ethylanthraquinone route in Figure 4(f), Pd_2_CO/Al_2_O_3_ was much more efficient than the atomically dispersed Pd_1_/TiO_2_-EG catalyst and also commercial Pd/C. The H_2_O_2_ yield on Pd_2_CO/Al_2_O_3_ was achieved as high as 93% with the production rate of 1054 g_H_2_O_2__ · g_Pd_^−1^ · h^−1^ (Table S8).

## 3. Discussion

To summarize, we demonstrate here that the coordination of small molecules on the ultrasmall metal clusters provides a powerful vector in tailoring their catalytic performance. For Pd clusters with a limited number of Pd atoms, such as Pd_2_ and Pd_3_, the too strong adsorption of the H atoms and alkyl radicals would inhibit the catalytic hydrogenation. Theoretical calculations predicted that hydrogenation activity would be significantly enhanced by introducing electron-withdrawing molecules, such as CO, on Pd clusters as the adsorption energies of hydrogen and hydrogenated intermediates were reduced. Supported Pd_2_CO clusters were successfully synthesized using dinuclear Pd-carbonyl clusters as the Pd precursor. Surprisingly, the mass-specific activity of supported Pd_2_CO exceeded those of the atomically dispersed Pd catalysts and Pd nanoparticles. The promotion effect of CO of the catalysis of small Pd clusters was unambiguously confirmed by removing and reintroducing CO. Our work reveals that the electronic and coordinative structures of metal clusters are significantly distinguished from those of the conventional metal nanoparticles.

## 4. Materials and Methods

Spin-polarized calculations were carried out with the Vienna ab initio simulation package (VASP) [49, 50]. The electron exchange and correlation were treated with the generalized gradient approximation using PBE functional [51]. The valence electrons were described by plane wave basis sets with a cut-off energy of 400 eV, and the core electrons were replaced by the projector augmented wave pseudopotential [52, 53]. Geometries of minima and TSs were converged to a residual force smaller than 0.03 eV/Å. The transition states were determined using the nudged elastic band (NEB) approach [54], with a subsequent quasi-Newton optimization to refine the TS' structures and energies. All the local minima and TSs were verified by vibrational frequency calculations.

For Pd_*n*_ clusters (*n* = 2, 3, 4, 7, 13, 55, and 147), as shown in Figure 1, the geometry structures with the highest symmetry were chosen [31, 55]. To avoid image interaction, the shortest distances between the image clusters were set to be more than 10 Å. In these cases, the Gamma point only calculations were performed. For the Pd(111) and Pd(100) surfaces, (3 × 4) supercells with five atomic layers were used. The vacuum regions between the slabs were 15 Å, and the *k*-point sampling was generated following the Monkhorst-Pack procedure with a 3 × 3 × 1 mesh. For the Pd surface models, the bottom two layers were fixed at a bulk truncated position, while the top three layers and the adsorbates were fully relaxed. For the Pd clusters on anatase, a five-layer TiO_2_(010) *p*(1 × 4) slab was used and the utermost surface was fully hydroxylated (Figure S1). The GGA + *U* approximation with the Dudarev “+*U*” term with a *U*‐*J* value of 4.2 eV for the d electrons of Ti atoms was adopted [56]. The structure of anatase-supported Pd_*n*_ was optimized by placing different initial configurations of Pd clusters on the surface. Such a method is appropriate to locate the global minima of ultrasmall clusters and has been frequently adopted in the literature [57, 58]. DFT calculations showed that not only the Pd_*n*_ cluster but also Pd_*n*_CO clusters (*n* = 2‐7) can strongly interact with the surface oxygen atoms over TiO_2_(010).

For the preparation of Pd_2_CO/TiO_2_, and Pd_2_CO/Al_2_O_3_, 10 *μ*L H_2_PdCl_4_ (1 M) was introduced into 1 mL THF in a glass bottle, and the solution was kept stirred under 0.2 MPa CO at room temperature till the color of the solution turned into bright yellow. Then, the solution was introduced dropwise into 20 mL THF dispersions of the supports (500 mg TiO_2_ or Al_2_O_3_) under stirring; then, the solvent was removed by centrifugation and dried under vacuum at room temperature; the as-obtained catalysts were denoted as Pd_2_CO/TiO_2_ and Pd_2_CO/Al_2_O_3_. The single-atom Pd catalysts were synthesized following the procedures reported previously [43, 44]. The colloidal Pd nanosheets (Pd NSs) and Pd nanocubes (Pd NCs) with preferential (111) and (100) exposed surface, respectively, were prepared following the procedures reported previously in our group [27, 41]. All these catalysts were used without any pretreatment. The 0.5 wt% Pd/Al_2_O_3_ was prepared following the typical impregnation method; the catalyst was calcined in air at 300°C for 2 h and reduced in H_2_ at 100°C for 1 h before applying in catalysis. The bridge site and hollow site CO adsorbed on 0.5 wt% Pd/Al_2_O_3_ after reduction suggested the presence of reduced Pd and Pd-Pd moieties on the surface.

For styrene hydrogenation, a proper amount of catalyst was introduced in 10 mL EtOH and stirred at 30°C and 0.1 MPa H_2_ atmosphere for 10 min; then, 0.55 mL (5 mmol) styrene was added. The ratio of substrate to catalyst (*S*/*C*) was controlled. For the gas-powder phase ethylene hydrogenation, Pd/Al_2_O_3_ was first reduced at 100°C for 30 min before cooled down to 30°C and applied in ethylene hydrogenation. For CO adsorption, the catalyst was treated with 5% CO/Ar (30 mL/min) at 30°C for 15 min, then flushed with feed gas at 60°C for 30 min before cooled down to 30°C. For CO desorption, the catalyst was treated with feed gas at 150°C for 30 min, then cooled down to 30°C again. The production of H_2_O_2_ was performed following the procedure reported in the literature [45].

## Data Availability

The data is available from the authors.

## Supplementary Materials

Supplementary Materials Supplemental information includes materials, additional experimental and theoretical methods, 41 figures, and 8 tables. Figure S1: optimized structure of anatase TiO_2_(010) surface. Figure S2: optimized structure of Pd_2_/TiO_2_, Pd_2_CO/TiO_2_, Pd_3_/TiO_2_, and Pd_3_CO/TiO_2_. Figure S3: optimized structure of Pd_4_/TiO_2_ and Pd_7_/TiO_2_. Figure S4: TS structures of Pd_2_/TiO_2_ and Pd_2_CO/TiO_2_-catalyzed styrene hydrogenation. Figure S5: reaction pathway for styrene hydrogenation on Pd_3_/TiO_2_ and Pd_3_CO/TiO_2_. Figure S6: TS structures of Pd_3_/TiO_2_ and Pd_3_CO/TiO_2_-catalyzed styrene hydrogenation. Figure S7: the optimized structures of 2H adsorption and coadsorption of CO and 2H on Pd(100) and Pd(111) surfaces. Figure S8: the optimized structures of Pd clusters with coadsorption of CO and 2H. Figure S9: the optimized structures of 2H adsorption and coadsorption of CO and 2H on Pd_4_/TiO_2_ and Pd_7_/TiO_2_. Figure S10: TS structures for H_2_ dissociation on Pd_2_CO/TiO_2_ and Pd_3_CO/TiO_2_. Figure S11: structures and adsorption energies of styrene on Pd_2_CO/TiO_2_ and Pd_2_/TiO_2_. Figure S12: the TEM images of TiO_2_-EG and TiO_2_. Figure S13: the N_2_ adsorption/desorption isotherm of the TiO_2_ and the XRD patterns of TiO_2_ before and after Pd_2_CO cluster deposition. Figure S14: the zeta potential and thermogravimetric analysis (TGA) of TiO_2_. Figure S15: the X-band EPR spectrum of the as-obtained TiO_2_ and Pd_2_CO/TiO_2_. Figure S16: low-magnification HAADF-STEM image and corresponding EDX element mapping of Pd_2_CO/TiO_2_. Figure S17: HRTEM images of Pd_2_CO/TiO_2_. Figure S18: UV-vis spectrum of the Pd_2_CO cluster and TiO_2_ powder before and after loading the Pd_2_CO cluster. Figure S19: HAADF-STEM images of Pd_2_CO/TiO_2_. Figure S20: the unit cell structure of (PPh_4_)_2_[Pd_2_(*μ*-CO)_2_Cl_4_]. Figure S21: the Pd K-edge XAS and XANES. Figure S22: EXAFS fitting of Pd foil, (PPh_4_)_2_[Pd_2_(*μ*-CO)_2_Cl_4_] and Pd_2_CO/TiO_2_. Figure S23: Releasing of CO_2_ upon adding TiO_2_ to the solution of H_2_[Pd_2_(*μ*-CO)_2_Cl_4_]. Figure S24: CO-DRIFTS of the as-obtained Pd_2_CO/TiO_2_. Figure S25: TPD-MS signal of CO for blank TiO_2_ and the as-obtained Pd_2_CO/TiO_2_. Figure S26: *in situ* DRIFTS for Pd_2_CO/TiO_2_ in ethylene hydrogenation. Figure S27: the mass-specific activity and TOF of styrene hydrogenation catalyzed by different Pd catalysts. Figure S28: isotopic experiment of Pd_2_CO/TiO_2_-catalyzed styrene hydrogenation. Figure S29: EXAFS fitting of Pd_2_CO/TiO_2_ after styrene hydrogenation. Figure S30: catalytic performance of Pd_2_CO/TiO_2_ in five test rounds. Figure S31: DRIFT spectrum of Pd_2_/TiO_2_-cal before and after treatment with CO. Figure S32: XANES and EXAFS fitting of Pd_2_/TiO_2_-cal after treatment with H_2_ and further with CO. Figure S33: catalytic performance and Arrhenius plot of Pd_2_/TiO_2_-cal-catalyzed styrene hydrogenation. Figure S34: catalytic styrene hydrogenation performance of Pd_1_/TiO_2_-cal and Pd cube with or without CO. Figure S35: the XRD pattern and N_2_ adsorption/desorption isotherm profile of *γ*-Al_2_O_3_. Figure S36: TEM images of 0.5 wt% Pd/Al_2_O_3_. Figure S37: H_2_ TPD, CO TPD, and H_2_ TPD with preadsorbed CO over 0.5 wt% Pd/Al_2_O_3_. Figure S38: the *in situ* CO-DRIFTS for 0.5 wt% Pd/Al_2_O_3_. Figure S39: TEM images of 0.2 wt% Pd_2_CO/Al_2_O_3_. Figure S40: EXAFS fitting of Pd_2_CO/Al_2_O_3_. Figure S41: styrene hydrogenation catalyzed by 0.2 wt% Pd_2_CO/Al_2_O_3_. Table S1: adsorption energies of 2H (Δ*E*_2H_) for Pd_*n*_ clusters on the TiO_2_(010) surface in the absence or in the presence of CO. Table S2: the Pd-Pd distances in Pd_*n*_ and Pd_*n*_CO clusters on TiO_2_(010) and the bare clusters. Table S3: the total magnetizations of Pd in Pd_*n*_ and Pd_*n*_CO clusters on TiO_2_(010) and the bare clusters. Table S4: the average bader charges of Pd in Pd_*n*_ and Pd_*n*_CO clusters on TiO_2_(010). Table S5: crystal data and structure refinement for (Ph_4_P)_2_[Pd_2_(*μ*-CO)_2_Cl_4_]. Table S6: atomic coordinates and equivalent isotropic displacement parameters for (Ph_4_P)_2_[Pd_2_(*μ*-CO)_2_Cl_4_]. Table S7: EXAFS fitting results. Table S8: production efficiency of the H_2_O_2_ following the 2-eAQ hydrogenation route catalyzed by different catalysts reported.
