# Supplementary material for: Carbon Monoxide Promotes the Catalytic Hydrogenation on Metal Cluster Catalysts
Source: Research (Wash D C). 2020 Jul 17;2020:4172794. doi: 10.34133/2020/4172794 (PMC7382763; doi:10.34133/2020/4172794)
Supplement: Supplementary Materials — Supplemental information includes materials, additional experimental and theoretical methods, 41 figures, and 8 tables. Figure S1: optimized structure of anatase TiO2(010) surface. Figure S2: optimized structure of Pd2/TiO2, Pd2CO/TiO2, Pd3/TiO2, and Pd3CO/TiO2. Figure S3: optimized structure of Pd4/TiO2 and Pd7/TiO2. Figure S4: TS structures of Pd2/TiO2 and Pd2CO/TiO2-catalyzed styrene hydrogenation. Figure S5: reaction pathway for styrene hydrogenation on Pd3/TiO2 and Pd3CO/TiO2. Figure S6: TS structures of Pd3/TiO2 and Pd3CO/TiO2-catalyzed styrene hydrogenation. Figure S7: the optimized structures of 2H adsorption and coadsorption of CO and 2H on Pd(100) and Pd(111) surfaces. Figure S8: the optimized structures of Pd clusters with coadsorption of CO and 2H. Figure S9: the optimized structures of 2H adsorption and coadsorption of CO and 2H on Pd4/TiO2 and Pd7/TiO2. Figure S10: TS structures for H2 dissociation on Pd2CO/TiO2 and Pd3CO/TiO2. Figure S11: structures and adsorption energies of styrene on Pd2CO/TiO2 and Pd2/TiO2. Figure S12: the TEM images of TiO2-EG and TiO2. Figure S13: the N2 adsorption/desorption isotherm of the TiO2 and the XRD patterns of TiO2 before and after Pd2CO cluster deposition. Figure S14: the zeta potential and thermogravimetric analysis (TGA) of TiO2. Figure S15: the X-band EPR spectrum of the as-obtained TiO2 and Pd2CO/TiO2. Figure S16: low-magnification HAADF-STEM image and corresponding EDX element mapping of Pd2CO/TiO2. Figure S17: HRTEM images of Pd2CO/TiO2. Figure S18: UV-vis spectrum of the Pd2CO cluster and TiO2 powder before and after loading the Pd2CO cluster. Figure S19: HAADF-STEM images of Pd2CO/TiO2. Figure S20: the unit cell structure of (PPh4)2[Pd2(μ-CO)2Cl4]. Figure S21: the Pd K-edge XAS and XANES. Figure S22: EXAFS fitting of Pd foil, (PPh4)2[Pd2(μ-CO)2Cl4] and Pd2CO/TiO2. Figure S23: Releasing of CO2 upon adding TiO2 to the solution of H2[Pd2(μ-CO)2Cl4]. Figure S24: CO-DRIFTS of the as-obtained Pd2CO/TiO2. Figure S25: [file 4172794.f1.pdf]

Supplementary Materials

for

Carbon Monoxide Promotes the Catalytic Hydrogenation on Metal Cluster Catalysts

Authors

Ruixuan Qin,[1]† Pei Wang,[1, 2]† Pengxin Liu,[1] Shiguang Mo,[1] Yue Gong,[3] Liting Ren,[1] Chaofa Xu,[1] Kunlong Liu,[1] Lin Gu,[3] Gang Fu,[1]* Nanfeng Zheng[1]*

Affiliations

[1] State Key Laboratory for Physical Chemistry of Solid Surfaces, Collaborative Innovation Center of Chemistry for Energy Materials, and National & Local Joint Engineering Research Center for Preparation Technology of Nanomaterials, College of Chemistry and Chemical Engineering, Xiamen University, Xiamen 361005, China.

[2] College of Science, Huazhong Agricultural University, Wuhan 430070, P. R. China

[3] Institute of Physics, Chinese Academy of Sciences, Beijing 100190, China.
† The authors contributed equally to this work

Correspondence should be addressed to Nanfeng Zheng; nfzheng@xmu.edu.cn and Gang Fu; gfu@xmu.edu.cn

Materials

Palladium chloride ($PdCl_2$, 59.8%), disodium tetrachloropalladate (Na_2PdCl_4) and palladium(II) acetate [$Pd(OAc)_2$] were purchased from Kunming Institute of Precious Metals. Titanium tetrachloride ($TiCl_4$), and 5% Pd/C were purchased from Alfa Aesar. Hydrochloric Acid (HCl ~35%), ethylene glycol (EG), tetrahydrofuran (THF), styrene, ethanol (EtOH), acetone, poly(vinylpyrrolidone) (PVP, MW= 55 000), L-ascorbic acid, dimethyl formamide (DMF), KBr 2-ethyl anthraquinone (2-eAQ), phosphoric acid, trioctyl phosphate (TOP), sulfuric acid and potassium permanganate were purchased from Sinopharm Chemical Reagent Co. Ltd. (Shanghai, China). Al_2O_3 was purchased from Hangzhou Wanjing New Materials Co. Ltd. (Hangzhou, Zhejiang, China) H_2 (99.99%), D_2 (99.99%), CO (99.95%) C_2H_4 (99.5%) and 5% CO/Ar were purchased from Linde Gas. The water was deionized water. All the chemicals were used without further purification (The commercial 5% Pd/C was reduced in 5% H_2/Ar at 100 °C for 1h before using).

Methods

<u>Preparation of TiO_2-EG and TiO_2</u>

Ultrathin TiO_2-EG nanosheets were synthesized corresponding to the reported method.[1] 2 mL $TiCl_4$ was carefully introduced into 60 mL EG and stirred under ultrasonic until homogeneous light yellow solution was obtained, then the solution was transferred into 100 mL Teflon-lined stainless-steel autoclave and heated at 150 °C for 4h. The obtained white colloids were centrifuged and washed with water for 3 times.

The EG-free TiO_2 that was obtained by heating TiO_2-EG at 350 °C for 2 h under air atmosphere in muffle with a ramp rate of 1 °C/min.

<u>Preparation of Pd_2CO/TiO_2, Pd_2/TiO_2-cal, Pd_2CO/Al_2O_3.</u>

10 μL H_2PdCl_4 (1 M) was introduced into 1 mL THF in a glass bottle, and the solution was kept stirred under 0.2 MPa CO at room temperature till the color of the solution turned into bright yellow. Then the solution was introduced dropwise into 20 mL THF dispersions of the supports (500 mg TiO_2 or Al_2O_3) under stirring, then the solvent was removed by centrifuge and dried under vacuum at room temperature, the as-obtained catalysts were denoted as Pd_2CO/TiO_2 and Pd_2CO/Al_2O_3.

The releasing of CO_2 upon introducing TiO_2 into the solution of $H_2[Pd_2(\mu\text{-}CO)_2Cl_4]$ was recorded by MX6 iBrid Gas Detector (Industrial Scientific) equipped with a CO_2 detector. The UV-vis spectrum of

the $H_2[Pd_2(\mu\text{-}CO)_2Cl_4]$/THF solution and as-obtained Pd_2CO/TiO_2 were performed in dilute and recorded by Shimadzu UV2600 instrument.

In order to remove the adsorbed CO, Pd_2/TiO_2-cal was obtained by heating Pd_2CO/TiO_2 at 350 °C for 1h (ramp rate, 2 °C/min) under static air atmosphere. Before applying in catalysis, Pd_2/TiO_2-cal was treated with H_2 at room temperature for 15 min.

Preparation of Pd_1/TiO_2-EG and Pd_1/TiO_2-cal

The single-atom dispersed Pd catalysts were synthesized following the procedures reported in our group previously.[2, 3] Typically, 200 mg TiO_2-EG nanosheets and 4 μL H_2PdCl_4 (1 M) were added in 20 mL H_2O and stirred at room temperature for 30 min. The colloidal solution was then irradiated under 365 nm filter equipped Xe-lamp (100 W) for 15 min. The colloids were centrifuged and washed with water for 2 times, then dried overnight under vacuum at room temperature. The obtained catalyst was denoted as Pd_1/TiO_2-EG. The EG-free single-atom dispersed Pd catalyst, Pd_1/TiO_2-cal was obtained by calcination of Pd_1/TiO_2-EG and at 350 °C for 4h (ramp rate, 1 °C/min) under static air atmosphere.

Preparation of Pd nanosheets and Pd nanocubes

The colloidal Pd nanosheets (Pd NSs) and Pd nanocubes (Pd NCs) with preferential (111) and (100) exposed surface respectively were prepared following the procedures reported previously in our group.[4, 5] For Pd nanosheets, 30 μL 1 M H_2PdCl_4 was added into 10 mL DMF and stirred under 0.1 MPa CO atmosphere at room temperature for 15 min, then 1 mL H_2O was introduced and stirred for another 15 min without CO. The colloidal Pd nanosheets were centrifuged and washed with EtOH and acetone for 3 times. For Pd nanocubes, 8 mL aqueous solution containing poly(vinylpyrrolidone) (PVP, MW= 55,000, 105 mg), L-ascorbic acid (60 mg), KBr (200 mg) were placed in a 20 mL vial, and pre-heated in air under magnetic stirring at 80 °C for 10 min. Then the aqueous solution of Na_2PdCl_4 (57 mg in 3 mL H_2O) was added into the vial and kept at 80 °C for another 3 h before it was cooled down to room temperature. The product was collected by centrifugation and washed with water-acetone mixture.

Preparation of 0.5 wt% Pd/Al_2O_3

200 mg Al_2O_3 was dispersed in 6 mL acetone, then $Pd(OAc)_2$/Acetone solution was introduced, the Pd mass loading was controlled to be 0.5 wt%. The samples dried at 40 °C overnight, the obtained

powders were then calcined (ramp rate, 2 °C/min) in static air at 300 °C for 2h. The catalysts were reduced in H_2 at 100 °C for 1 h before applied in hydrogenation.

Catalysis tests

For styrene hydrogenation, a proper amount of catalyst was introduced in 10 mL EtOH and stirred at 30 °C and 0.1 MPa H_2 atmosphere for 10 min, then 0.55 mL (5 mmol) styrene was added and started to measure the conversion of styrene on a KB-WAX chromatographic column equipped gas chromatograph (GC). The ratio of substrate to catalyst (S/C) was controlled, for 0.2 wt% Pd_2CO/TiO_2 Pd_2/TiO_2-cal, Pd_1/TiO_2, Pd_1/TiO_2-cal and Pd nanosheets S/C=50,000. The activation energy was measured by adjusting the temperature to 20 °C 30 °C, 40 °C and 50 °C. In order to evaluate the effect of the different amount of CO on Pd_2/TiO_2-cal, Pd_1/TiO_2-EG and Pd_1/TiO_2-cal, different amount of dilute CO was introduced to the bottle before styrene hydrogenation was carried out. The effect of CO on the colloid Pd NSs and Pd NCs was evaluated by soaking them in 5% CO/Ar in EtOH for 15 min and then flushing with H_2 for 15 min, then styrene was introduced. The mass specific activity was calculated based on the total amount of Pd in the corresponding catalyst as a conversion level lower than 20%.

For the gas-powder phase ethylene hydrogenation. 2 mg 0.5 wt% Pd/Al_2O_3 diluted with 20 mg Al_2O_3 was loaded in a glass tube (8 mm diameter). The sample was reduced at 100 °C with 30 mL/min H_2 for 30 min before cooled down to 30 °C. Then, the catalytic hydrogenation was carried out with the feed gas flow of 25 mL/min C_2H_4 and 75 mL/min H_2, the conversion of C_2H_4 was determined by on-line GC. For CO adsorption, the catalyst was treated with 5% CO/Ar (30 mL/min) at 30 °C for 15 min, then flushed with the feed gas at 60 °C for 30 min before cooled down to 30 °C. For CO desorption, the catalyst was treated with feed gas at 150 °C for 30 min, then cooled down to 30 °C again.

The production of H_2O_2 was performed following the procedure reported in the literature.[6] Typically, 100 mg 0.2 wt% Pd_2CO/Al_2O_3 were dispersed in 10 mL TOP and then 2.4 g 2-eAQ diluted in 10 mL toluene were added. The hydrogenation of 2-eAQ was performed under 0.2 MPa H_2 at 30 °C. 1 mL of the solution was taken out and introduced into 10 mL of 1 M H_3PO_4 every 30 min, the obtained liquid was stirred in air for 30 min at room temperature. 5 mL of 20 % H_2SO_4 was added before the amount of H_2O_2 was titrated by 0.02 M $KMnO_4$.

Characterizations

Transmission Electron Microscope (TEM) characterization

For TEM characterization, the samples were dispersed in EtOH and dropping onto 300-mesh carbon-coated copper grids and the solvent was evaporated in air subsequently. TEM characterization and energy dispersive X-ray spectroscopy (EDX) was carried out on a TECNAI F30 transmission electron microscope operating at 300 kV.

HAADF-STEM characterizations

High-resolution transmission electron micrographs (HRTEM) were performed on JEOL 200F transmission electron microscope operated at 200 keV. Both annular-bright-field (ABF) and high-angle annular-dark-field (HAADF) images were acquired with the illumination semi-angle of 25 mrad and probe current of 100 pA. The dwell time for image acquisition was set at 10 micro second per pixel to ensure desirable signal to noise ratio. The attainable spatial resolution of microscope was 78 pm with a probe spherical-aberration corrector. The collection angles for the ABF and HAADF images were fixed at 12-25 mrad and 90-250 mrad, respectively.

Powder X-ray diffraction (XRD) characterizations

The XRD experiments were carried out on Rigaku Ultima IV using Cu Kα radiation. The operation voltage was set at 40 kV, the current was set at 30 mA. The scan speed was set at 10 °/min

Zeta-potential characterizations

Zeta-potential experiments were tested on Nano-ZS zetasizer (Malvern Instruments, UK). 2.0 mg TiO_2 was dispersed into 10 mL water with varied pH value (2 – 12) and 3 times tests were repeated for every pH point.

N_2 adsorption and desorption experiments.

The BET (Brunauer-Emmett-Teller) surface area of oxides was measured by N_2 adsorption-desorption experiments on Micromeritics ASAP2020 at liquid nitrogen temperature. The samples were degassed at 200 °C for 3h.

Temperature Programmed Desorption-mass spectrometry (TPD-MS) characterization

The TPD-MS experiment was performed on a home-made TPD-TOF analyzer. 3 mg of 1 wt% Pd_2CO/TiO_2 was added into a small tube which will be heated by the surrounded heating coil. A K-

type thermocouple was put inside the sample tube, and insulated from samples to measure the temperature. The heating coil was powered by a precise electric source, and adjusted at interval of 10 mV. The temperature of sample tube was ramping from room temperature to 450 °C with a rate of 5 K/min. The desorbed spices were ionized by a UV lamp at position very close to the sample tube, with phonon energy of 10.6 eV, and then transferred to TOF analyzer by an ion optical system. The TOF analyzer had a resolution of more than 5000, and the sensitivity of ppb level. All those steps were processed in high vacuum of about 3×10^{-5} Pa. The mass spectrum and sample temperature were acquired and recorded every second. Each spectrum was an accumulation of 10000 spectra gathered at interval of 100 μs.

X-ray absorption spectroscopy (XAS) measurements and data processing

The X-ray absorption experiments were carried out at the XAS station (BL14W1) of the Shanghai Synchrotron Radiation Facility (SSRF). The electron storage ring was operated at 3.5 GeV. Si(311) double-crystal was used as the monochromator, and the data was collected using solid-state detector under ambient conditions. The beam size was limited by the horizontal and vertical slits with the area of 1×4 mm^2 during XAS measurements. The X-ray absorption of Pd foil at Pd K-edge of was measured for energy calibration. All the samples were sealed in N_2 atmosphere before taking to the station, and the data was recorded under air atmosphere. The obtained XAFS data was processed in Athena (version 0.9.25) for background, pre-edge line and post-edge line calibrations. Then Fourier transformed fitting was carried out in Artemis (version 0.9.25).[7] The k^2 weighting, k-range of ~3 - 12 $Å^{-1}$ and R range of 1-3 Å were used. The model of bulk Pd and PdO were used to calculate the simulated scattering paths. The coordination number of Pd-Pd for Pd foil was fixed at 12 to determine the amplitude reduction factor (S_0^2=0.87). Then the four parameters, coordination number, bond length, Debye-Waller factor and E_0 shift (CN, R, σ^2, ΔE_0) were fitted without anyone was fixed, constrained, or correlated.

Pd dispersion determination

Pd dispersion was measured by CO titration carried out on a Micromeritics Auto Chem II 2920 chemical adsorption instrument equipped with a TCD detector. The samples were reduced at 100 °C in H_2 for 1h before cooled down to 50 °C. Then flashed with He for 1h and CO titration was performed with 5% CO/He. The Pd dispersion was calculated based on the consumed CO molecules and ratio between Pd and CO was 2:1.

Diffuse reflectance infrared Fourier transform spectroscopy (DRIFTS)

DRIFTS was carried out on ThermoFisher IS50 Fourier Transform Infrared spectrometer equipped with MCT detector. For Pd_2CO/TiO_2, the spectrum was recorded under ambient condition, and KBr powder was used as the background. For 0.5 wt% Pd/Al_2O_3, the sample were loaded in an *in-situ* chamber (Harrick) and treated with 5% H_2/Ar (30 mL/min) at 100 °C for 1 h, then cooled down to room temperature and the background spectrum was collected before treated with 5% CO/Ar for 20 min. The chamber was flushed with Ar for 20 min. The chamber was heated up to 30, 60 and 100 °C with 25 mL/min C_2H_4 and 75 mL/min H_2 for ~10 min, then cooled down to room temperature and flushed with Ar for 10 min before recording. For Pd_2/TiO_2-cal, after flushing with Ar and recording the background spectrum, the sample was treated with 5% CO/Ar for 20 min and then flushing with Ar before recording the spectrum.

Electron Paramagnetic Resonance (EPR)

X-band EPR spectra were recorded by a Bruker EMX-10/12 microspectrometer at 90 K, with an operation frequency of 9.45 GHz and a microwave power of 19.9 mW. In a typical measurement, 50 mg sample was used.

Temperature programed desorption

AutoChem 2920 II equipped with TCD detector was used to perform these measurements. For H_2-TPD, 0.5 wt% Pd/Al_2O_3 was first reduced with 5% H_2/Ar at 100 °C for 1h after calcined in static air. The sample was pretreated under 200 °C with Ar flow for 1 h before cooling down to 50 °C. The sample was further treated with 5% H_2/Ar and Ar for 30 min before the recording, respectively. CO-TPD was performed following a similar procedure except 5% CO/Ar was applied after cooling down to 50 °C. For the H_2-TPD with pre-adsorbed CO, the sample treated with 5% CO/Ar and 5% H_2/Ar for 30 min stepwise before flushing with Ar and recording.

Computational details

Spin-polarized calculations were carried out with the Vienna ab initio simulation package (VASP).[8, 9] The electron exchange and correlation were treated with the generalized gradient approximation using PBE functional.[10] The valence electrons were described by plane wave basis sets with a cut-off energy of 400 eV, and the core electrons were replaced by the projector augmented wave pseudopotential.[11, 12] Geometries of minima and transition states (TSs) were converged to a

residual force smaller than 0.03 eV/Å. The TSs were determined using the nudged elastic band (NEB) approach,[13] with a subsequent quasi-Newton optimization to refine the TS' structures and energies. All the local minima and TSs were verified by vibrational frequency calculations.

For Pd_n clusters (n=2, 3, 4, 7, 13, 55, 147), the geometry structures with the highest symmetry were chosen,[14, 15] as shown in Figure 1. To avoid image interaction, the shortest distances between the image clusters were set to be more than 10 Å . In these cases, the Gamma point only calculations were performed. For the Pd(111) and Pd(100) surfaces, (3×4) supercells with five atomic layers were used. The vacuum regions between the slabs were set to 15 Å, and the k-points sampling was generated following the Monkhorst-Pack procedure with a 3×3×1 mesh. For the Pd surface models, the bottom two layers were fixed at a bulk truncated position, while the top three layers and the adsorbates were allowed to be fully relaxed.

For the practical use, the Pd clusters would be loaded on the oxide surfaces, such as anatase TiO_2(010). Computationally, to model the anatase TiO_2 (010) surface, a five-layer p(1×4) slab was used and the utmost surface was fully hydroxylated (Figure S1). Since GGA was not able to correctly describe the electronic structure of Ti^{4+}, we adopted the GGA+U approximation with the Dudarev ''+U'' term with a U-J value of 4.2 eV for the d electrons of Ti atoms.[16] The binding energies of the Pd_n and Pd_nCO cluster on TiO_2(010) surface were calculated via $\Delta E_{bind} = E_{cluster/TiO2(010)} - E_{cluster} - E_{TiO2(010)}$, where $E_{cluster/TiO2(010)}$ denoted the energies of supported clusters, $E_{cluster}$ represented the energies of free clusters and $E_{TiO2(010)}$ stood for the energy of TiO_2(010) surface. DFT calculations showed that not only the Pd_n clusters but also Pd_nCO clusters (n=2-7) could strongly interact with the surface oxygen atoms over TiO_2 (010) with the ΔE_{bind} of -1.64eV~-2.95 eV.

The adsorption energy (ΔE_{2H}) were defined as $\Delta E_{2H} = E_{2H/surf} - E_{H2} - E_{surf}$. Here $E_{2H/surf}$, E_{H2}, and E_{surf} represented the energies of the surfaces with two adsorbed H atoms, molecular H_2 and the clean surface, respectively. The reaction energy (ΔE) for styrene hydrogenation was defined as $\Delta E = E_{ads/surf} - E_{PhCHCH2} - E_{2H/surf}$, where the sum of the energies of H preadsorbed surface ($E_{2H/surf}$) and molecular styrene ($E_{PhCHCH2}$) was used as energy reference. The activation barrier for each elementary step was defined as $\Delta E_a = E_{TS} - E_R$, where E_R and E_{TS} were the energies of the reaction intermediate and the corresponding transition state, respectively.

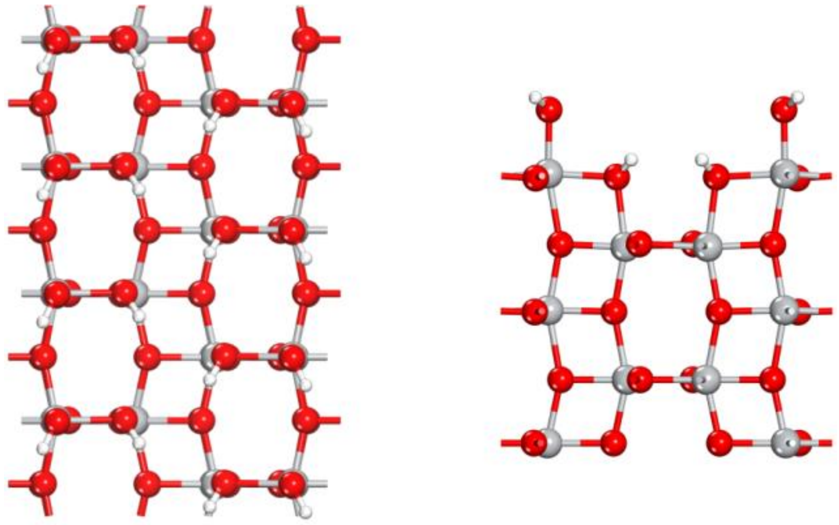

Figure S1. Top view (left) and side view (right) of the optimized structure of anatase TiO_2(010) surface. The light gray, red and white balls represented Ti, O and H atom, respectively.

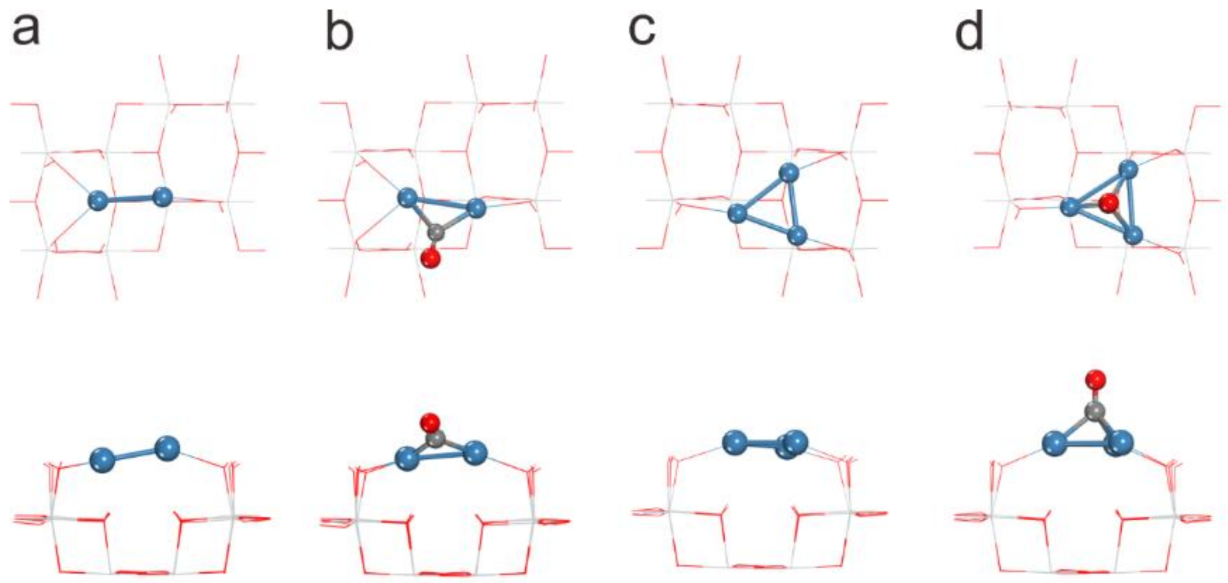

Figure S2. Top view (top) and side view (bottom) of the optimized structure of (a) Pd_2/TiO_2, (b) Pd_2CO/TiO_2, (c) Pd_3/TiO_2, and (d) Pd_3CO/TiO_2. The blue, dark gray, red and white balls represented Pd, C, O and H atom, respectively.

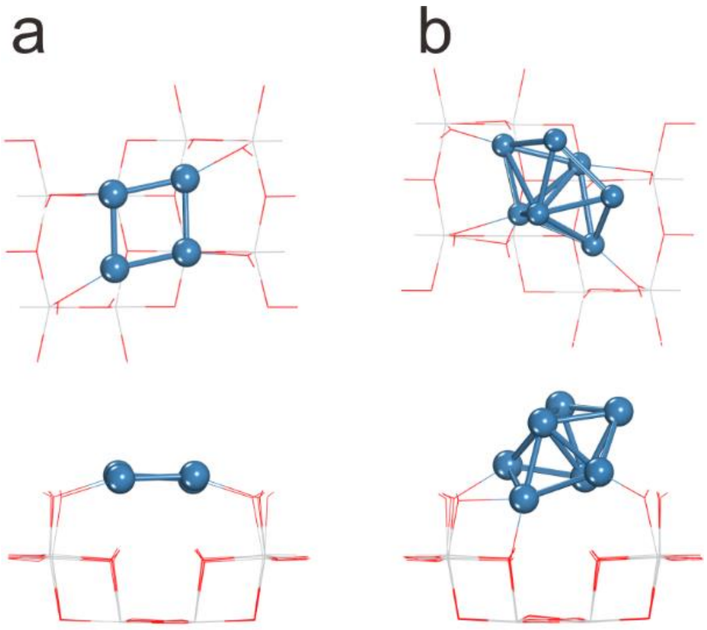

Figure S3. Top view (top) and side view (bottom) of the optimized structure of (a) Pd_4/TiO_2, (b) Pd_7/TiO_2. The blue, dark gray, red and white balls represented Pd, C, O and H atom, respectively.

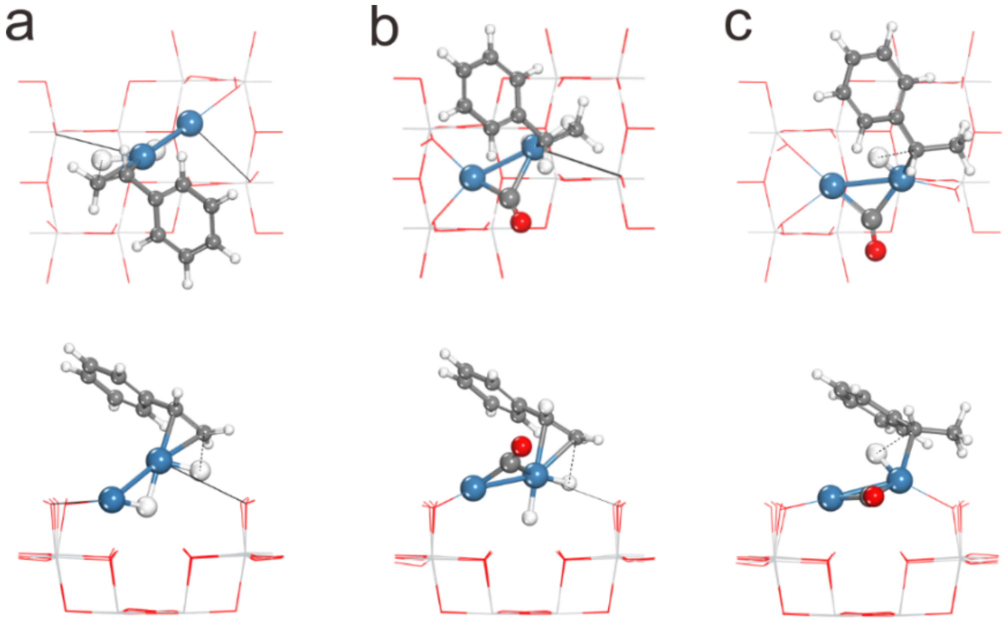

Figure S4. Top view (top) and side view (bottom) of the TS structures along the reaction path for (a) TS1 on Pd_2/TiO_2, (b)TS1 and (c)TS2 on Pd_2CO/TiO_2, respectively. The blue, dark gray, red and white balls represented Pd, C, O and H atom, respectively.

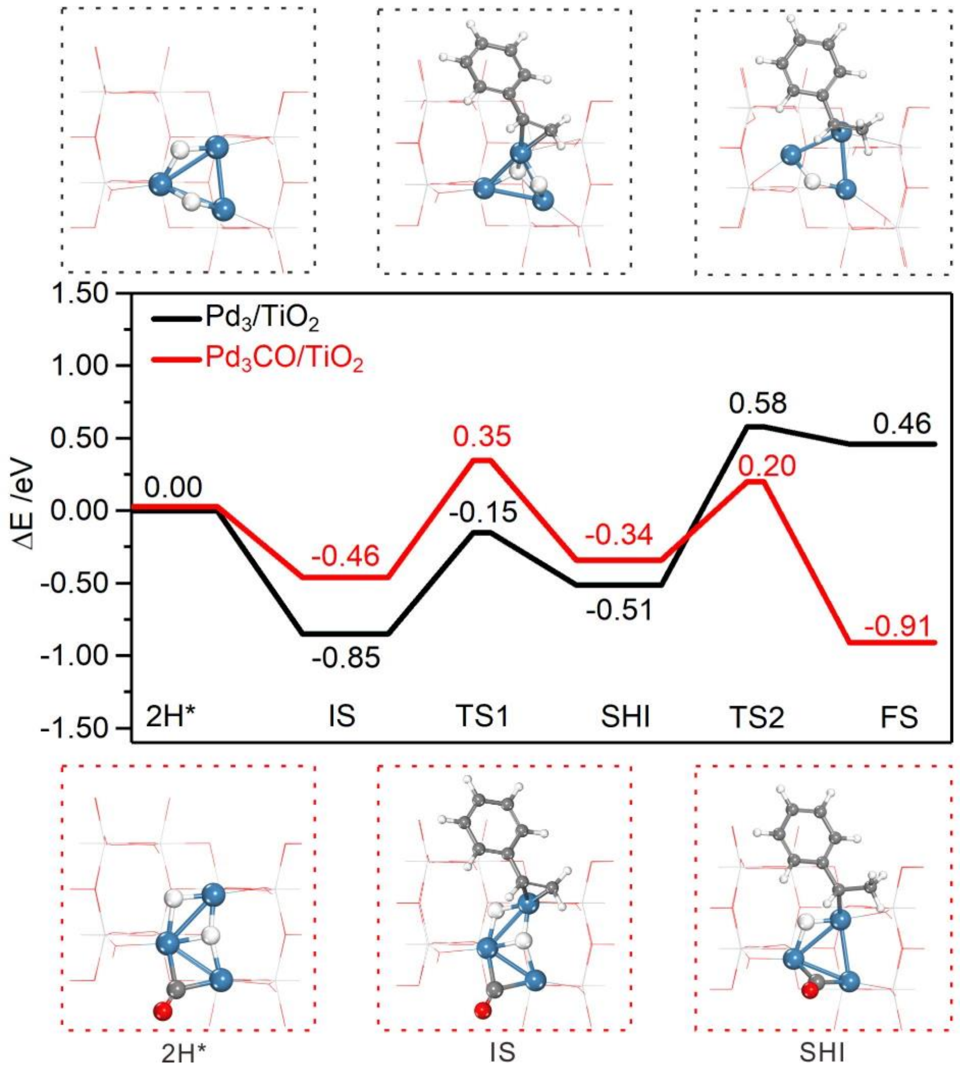

Figure S5. Reaction pathway for styrene hydrogenation on Pd_3/TiO_2 (black line) and Pd_3CO/TiO_2 (red line) surface. The optimized structures of key intermediates were illustrated in the dash line frame. The blue, red, dark gray and white balls represented Pd, O, C and H atom, respectively.

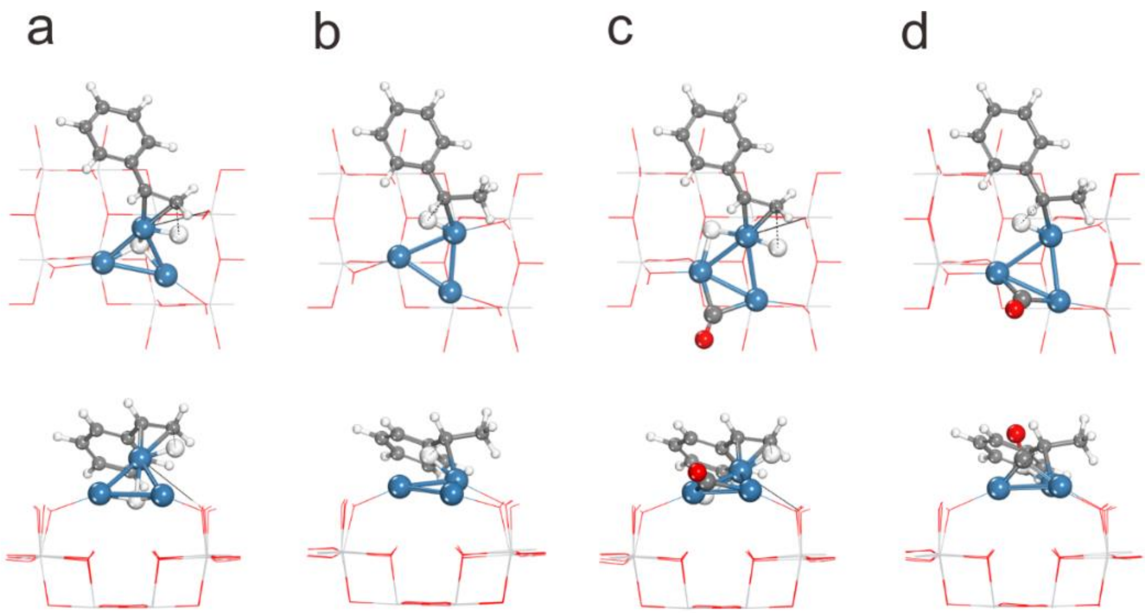

Figure S6. Top view (top) and side view (bottom) of the TS structures along the reaction path for (a) TS1 and (b) TS2 on Pd_3/TiO_2, (c) TS1 and (d) TS2 on Pd_3CO/TiO_2. The blue, dark gray, red and white balls represented Pd, C, O and H atom, respectively

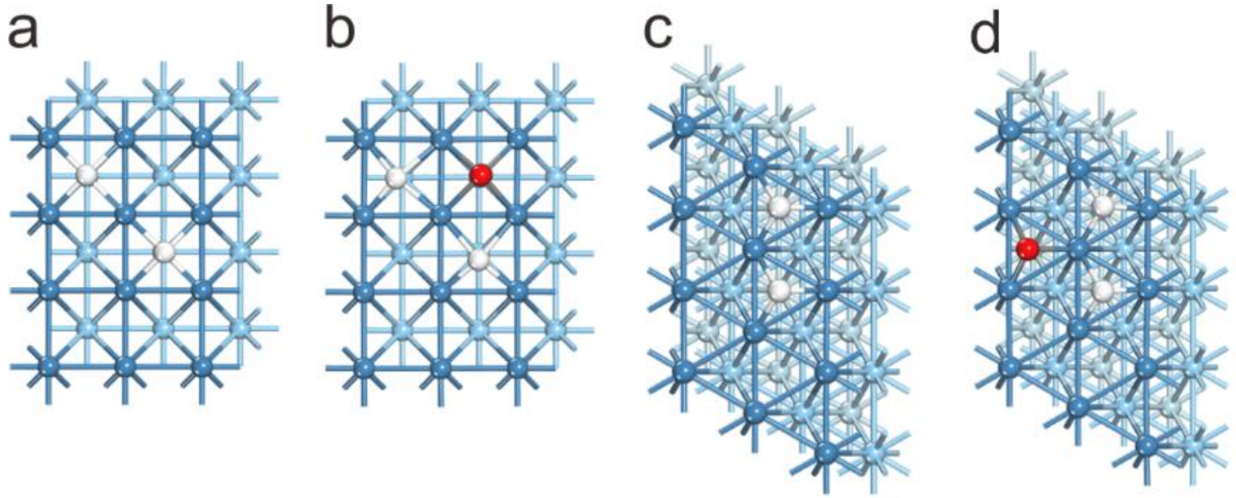

Figure S7. The optimized structures of (a, c) 2H adsorbed and (b, d) co-adsorption of CO and 2H neighbor to CO over (a, b) Pd(100) surface and (c, d) Pd(111) surface, respectively. The blue, dark gray, red and white balls represented Pd, C, O and H atom, respectively

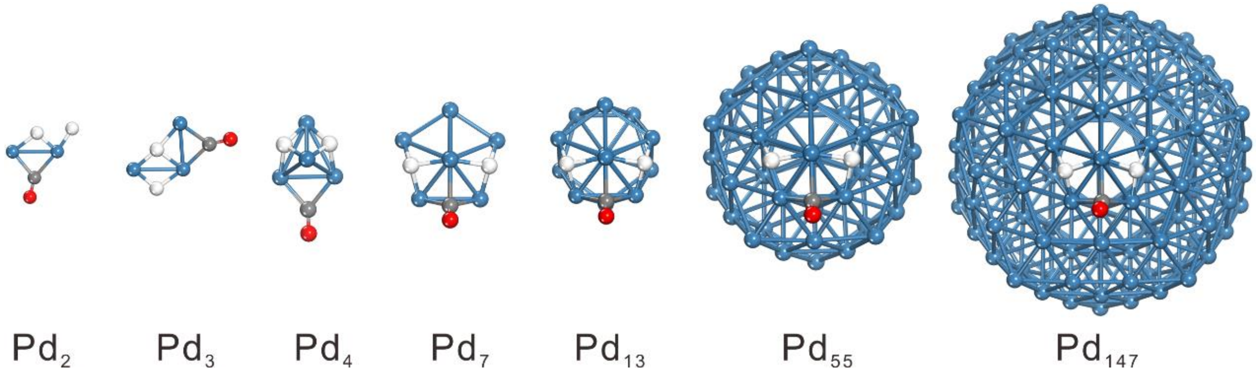

Figure S8. The optimized structures of Pd clusters (Pd_2, Pd_3, Pd_4, Pd_7, Pd_{13}, Pd_{55} and Pd_{147}) with co-adsorption of CO and 2H. The blue, red, dark gray and white balls represented Pd, O, C and H atom, respectively.

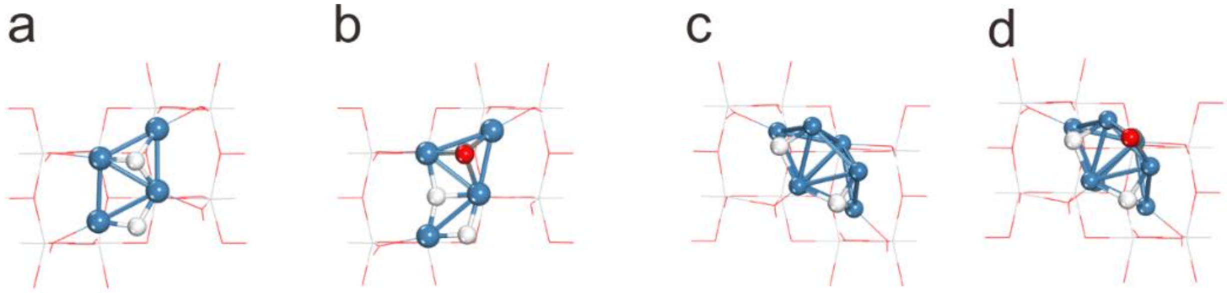

Figure S9. The optimized structures of (a, c) 2H adsorbed and (b, d) co-adsorption of CO and 2H neighbor to CO adsorbed Pd_4/TiO_2 and Pd_7/TiO_2, respectively. The blue, red, dark gray and white balls represented O, C and H atom, respectively.

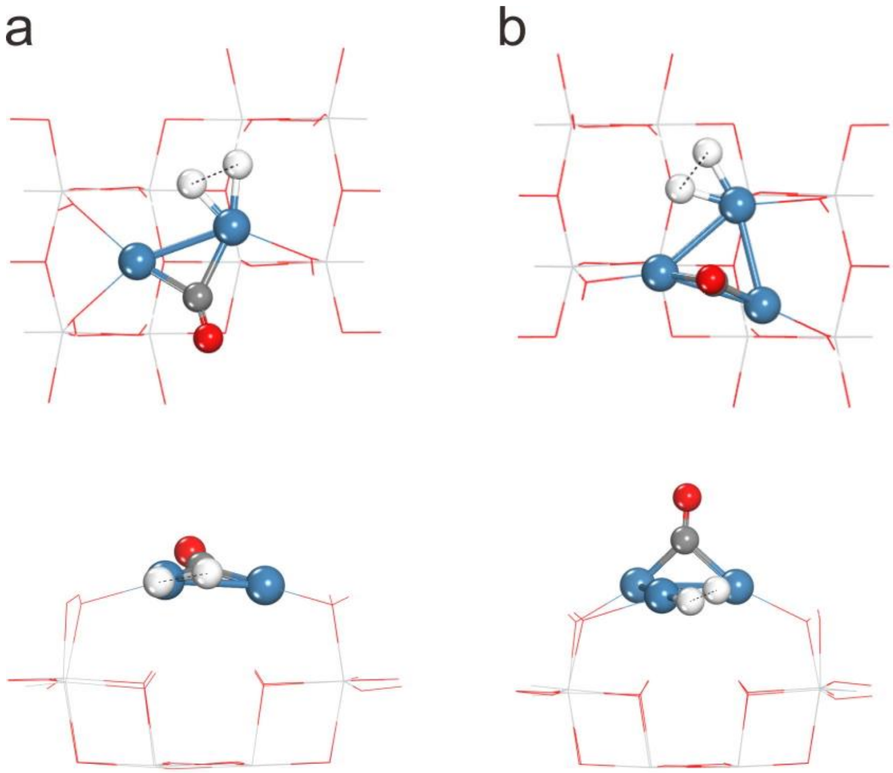

Figure S10. Top view (top) and side view (bottom) of the TS structures for H_2 dissociation on (a) Pd_2CO/TiO_2 and (b) Pd_3CO/TiO_2. The blue, dark gray, red and white balls represented Pd, C, O and H atom.

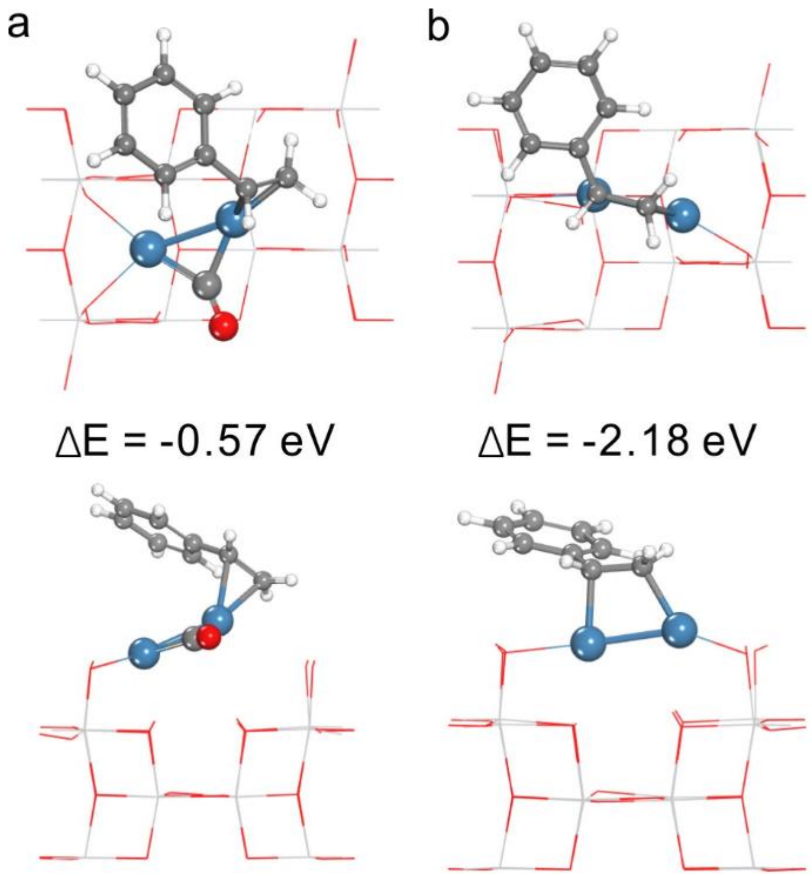

Figure S11. Top view (top) and side view (bottom) of styrene adsorbed on (a) Pd_2CO/TiO_2 and (b) Pd_2/TiO_2, and the corresponding adsorption energy, respectively. The blue, dark gray, red and white balls represented Pd, C, O, and H atom, respectively.

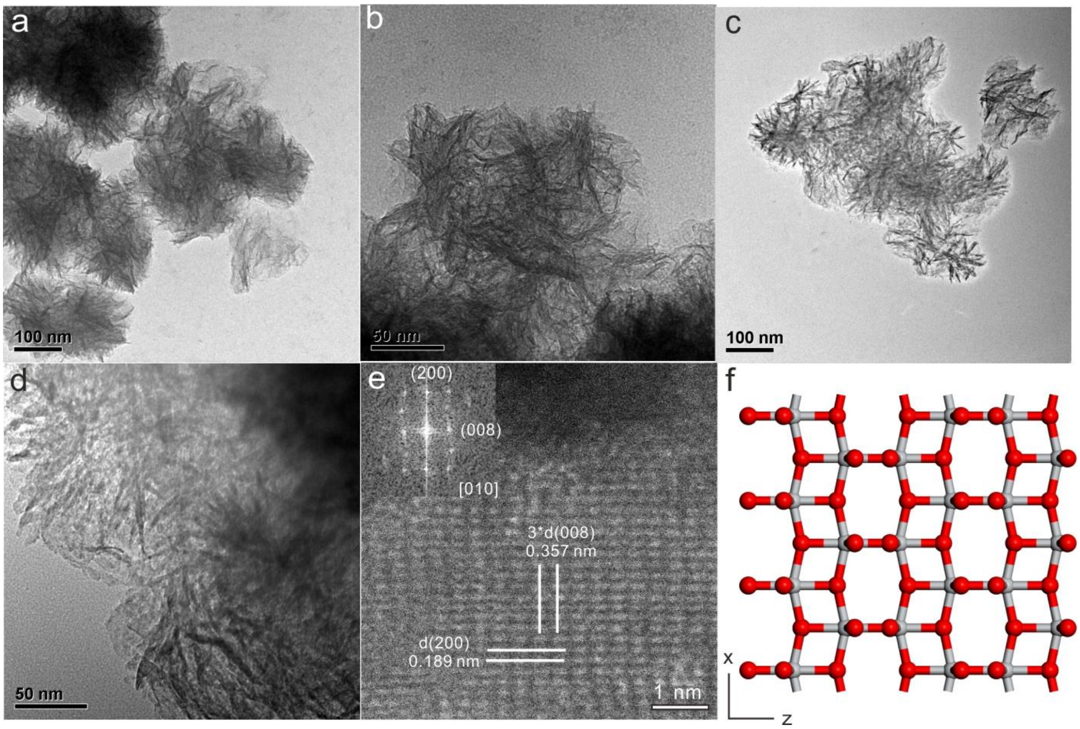

Figure S12. The TEM images of TiO_2-EG (a, b), and TiO_2 (c & d). (e) The HRTEM of TiO_2 showed the exposed (010) facet, which was consistent with the reported work.[2, 3] (f) The top view of anatase (010) facet.

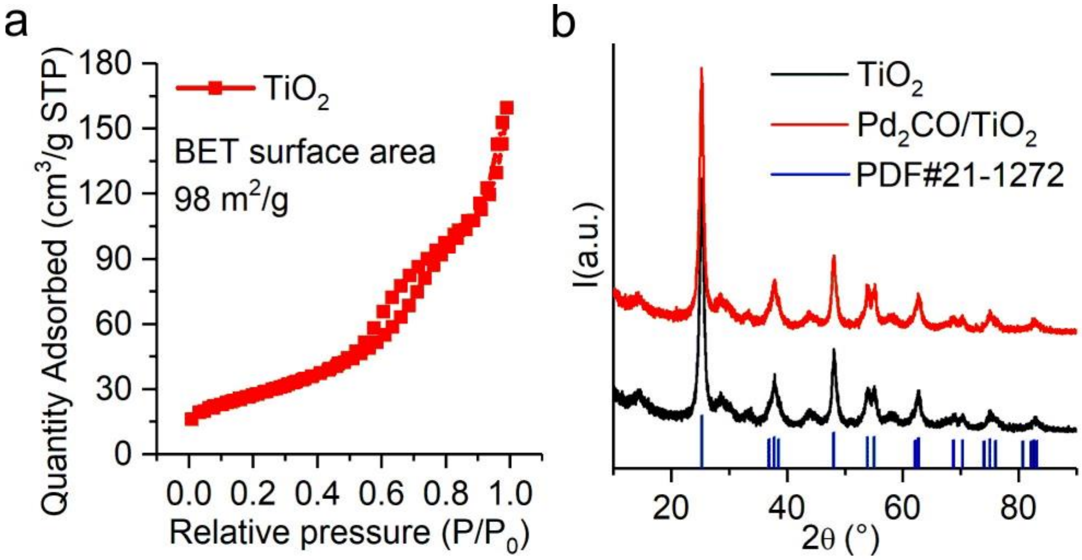

Figure S13. (a) The N_2 adsorption/desorption isotherm of the TiO_2 at 77K, the sample was degassed at 200 °C for 3 h. (b) The XRD patterns of support before and after Pd_2CO cluster deposition. No change was observed after deposition. The XRD pattern revealed the anatase phase of the support.

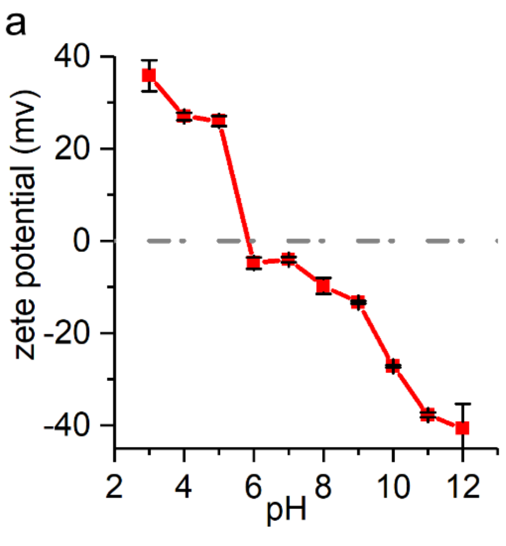

Figure S14. (a) The zeta-potential of the TiO_2 dispersed in water with different pH values. The isoelectronic point was about 6, therefore, the negatively charged $[Pd_2(\mu\text{-}CO)_2Cl_4]^{2-}$, would be easily adsorbed. (b) Thermogravimetric analysis (TGA) of TiO_2. The ~2.5% weight loss was ascribed to the dehydration of surface adsorbed H_2O and the surface –OH groups, which could react with $[Pd_2(\mu\text{-}CO)_2Cl_4]^{2-}$.

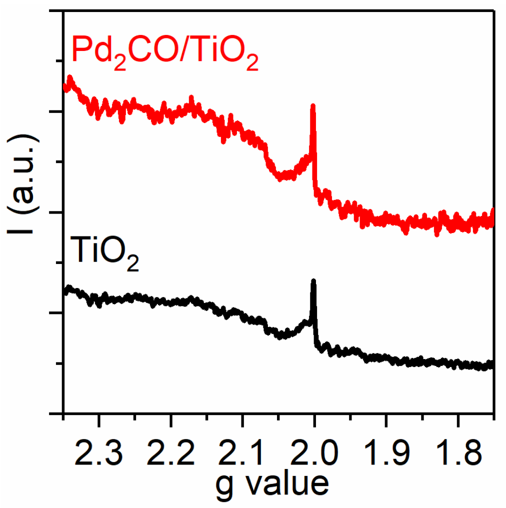

Figure S15. The X-band EPR spectrum of the as-obtained TiO_2 and Pd_2CO/TiO_2. No apparent change was observed before and after deposition of Pd_2CO cluster. The small and sharp signal with a g tensor about 2.00 was related to the trapped interior electrons or Ti^{3+}.[17, 18] No significant surface vacancies were observed.

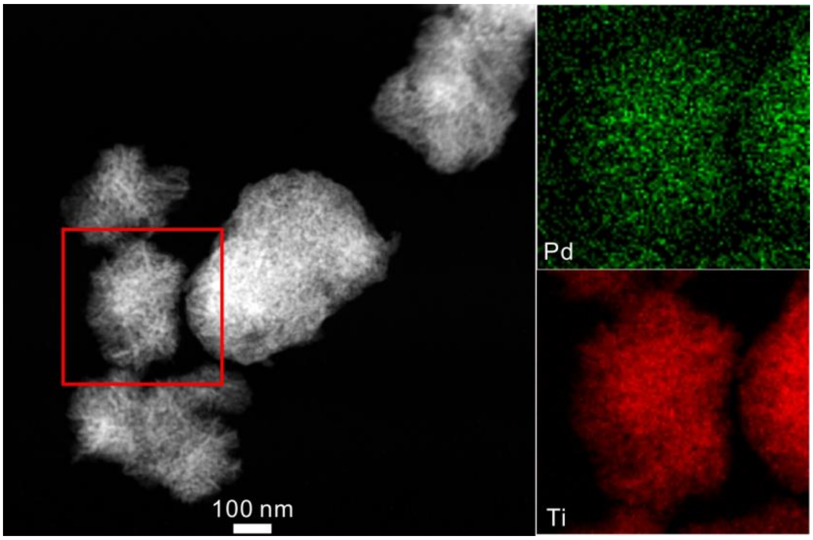

Figure S16. Low magnification HAADF-STEM image and corresponding EDX elements mapping of Pd_2CO/TiO_2, indicating that palladium was well dispersed on TiO_2.

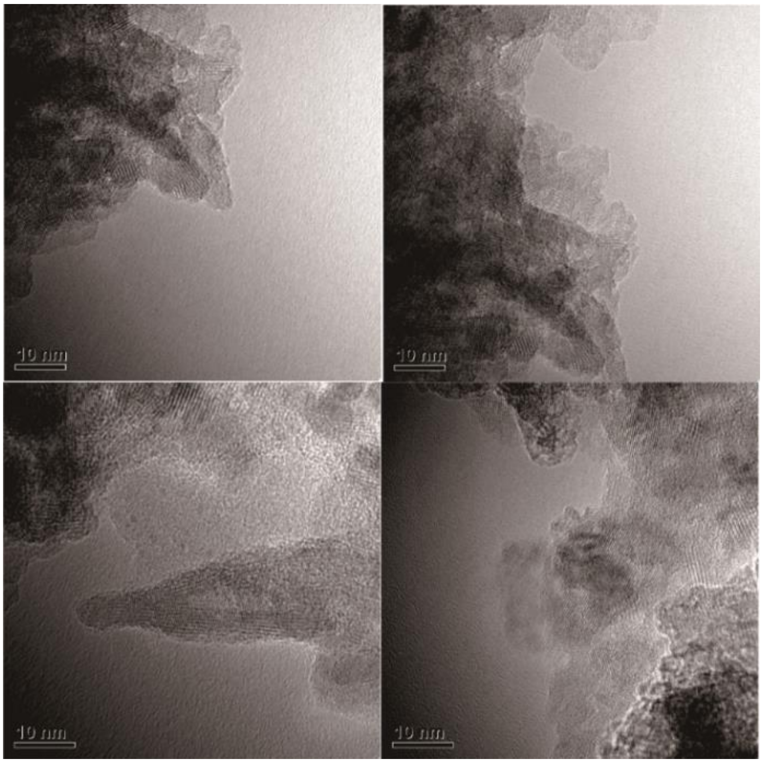

Figure S17. HRTEM images of Pd_2CO/TiO_2. No Pd nanoparticle was figured out on TiO_2.

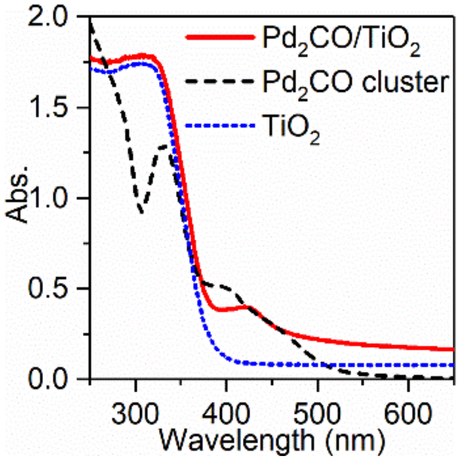

Figure S18. UV-vis spectrum of the Pd_2CO cluster (i.e. $H_2[Pd_2(\mu\text{-}CO)_2Cl_4]$ in THF) and TiO_2 powder before and after loading the Pd_2CO cluster. The UV-vis spectrum of the Pd_2CO cluster with wavelength below 350 nm was related to the metal-ligand electron transfer. Unfortunately, this feature was overlapped with the spectrum of TiO_2 at this region (the presence of CO was verified by the DRIFTS in Figure S23). The peaks with wavelength over 400 nm were related to the metal-metal d-d electron transfer.[19, 20] The wavelength of this feature is related to the nuclearity/size of the cluster and the distance between Pd atoms. The predominant peak at ~420 nm indicated that the small Pd clusters Pd_nCO with n close to 2-3 were dominated in the as-obtained catalyst.[21, 22]

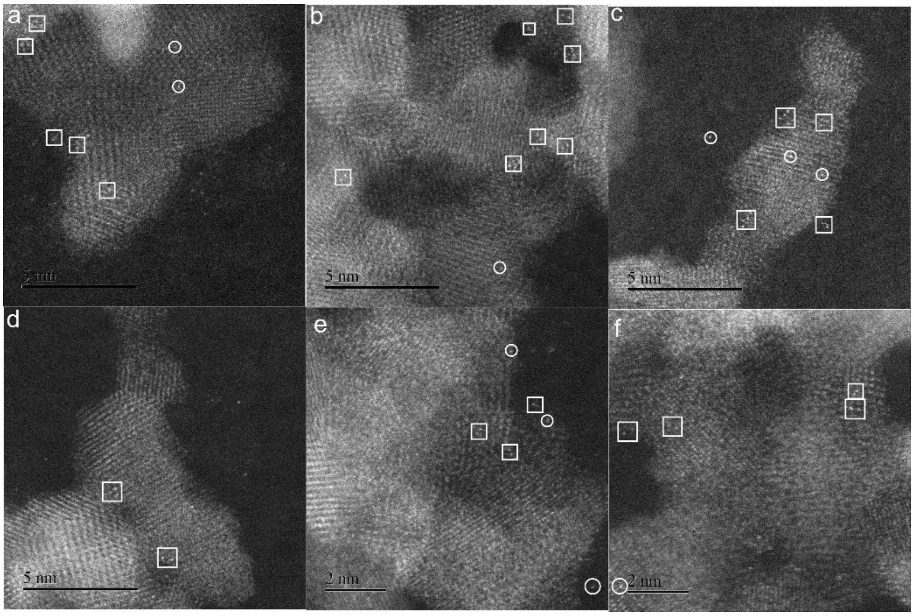

Figure S19. HAADF-STEM images of Pd_2CO/TiO_2, the white squire indicates the Pd cluster, and white circle for the single-atom Pd.

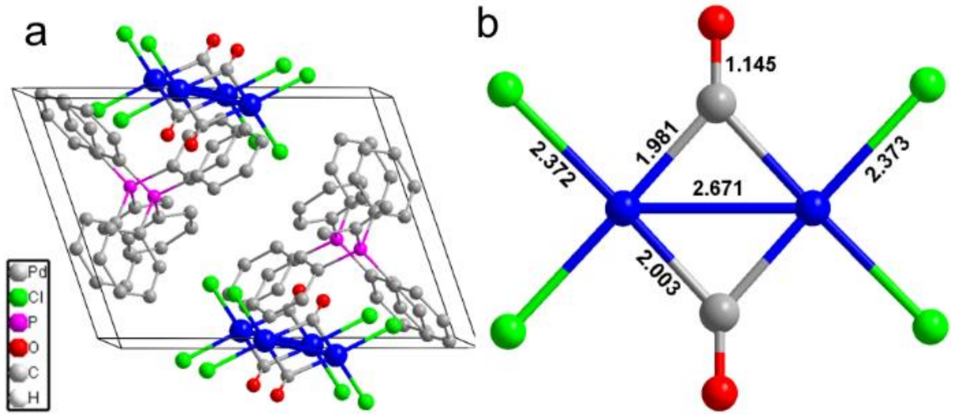

Figure S20. (a) The unit cell of $(PPh_4)_2[Pd_2(\mu\text{-}CO)_2Cl_4]$. (b) Bond lengths (Å) in $[Pd_2(\mu\text{-}CO)_2Cl_4]^{2-}$.

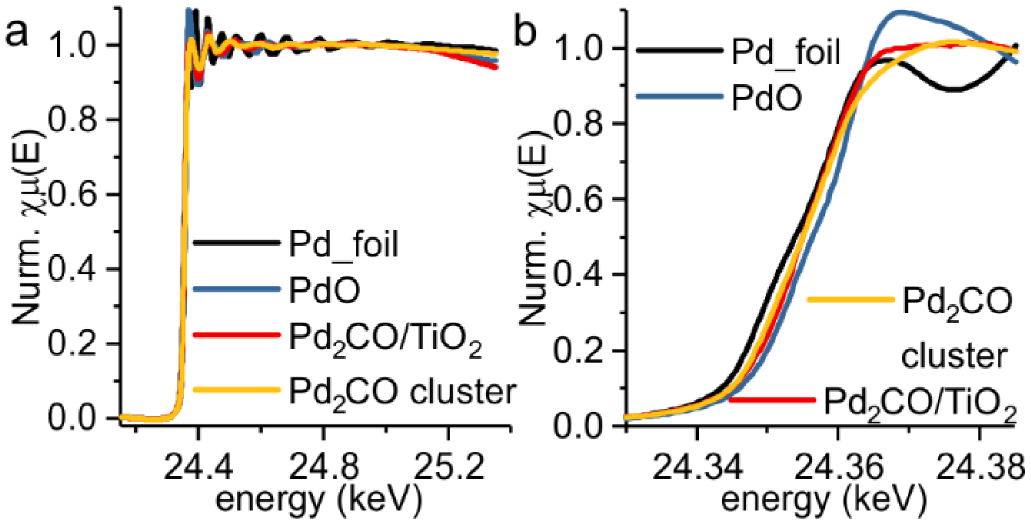

Figure S21. The Pd K-edge (a) XAS and (b) XANES of Pd foil, PdO, $(PPh_4)_2[Pd_2(\mu\text{-}CO)_2Cl_4]$ crystal and Pd_2CO/TiO_2.

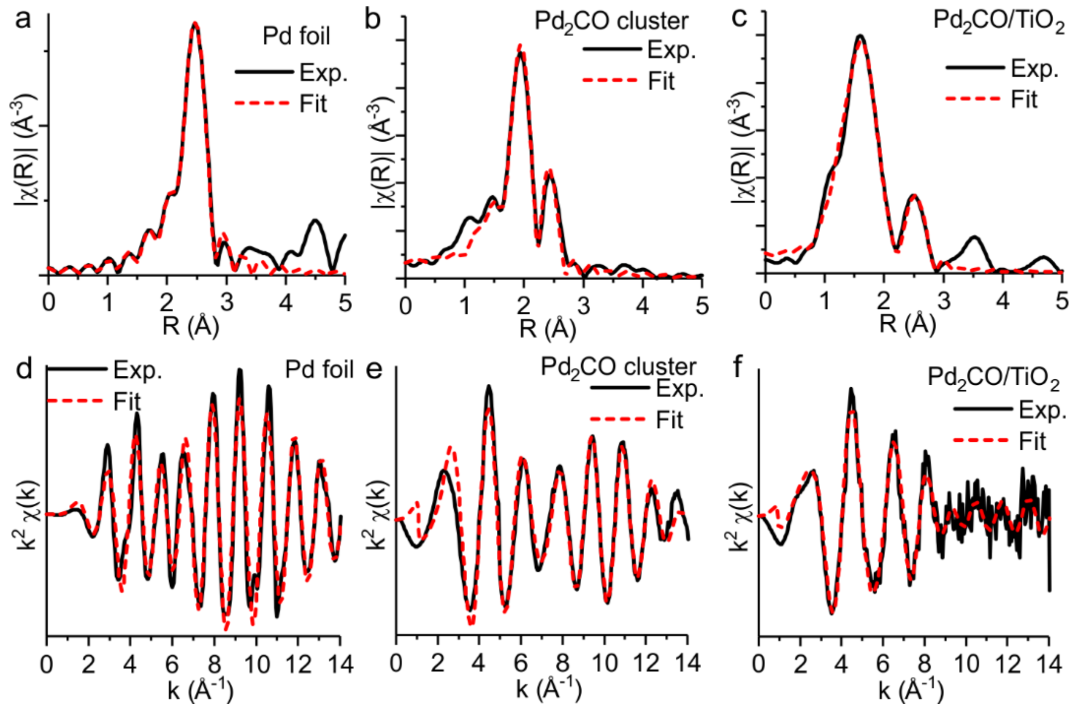

Figure S22. EXAFS (a-c) R-space and (d-f) k-space fitting of (a, d) Pd foil, (b, e) $(PPh_4)_2[Pd_2(\mu\text{-}CO)_2Cl_4]$ and (c, f) Pd_2CO/TiO_2.

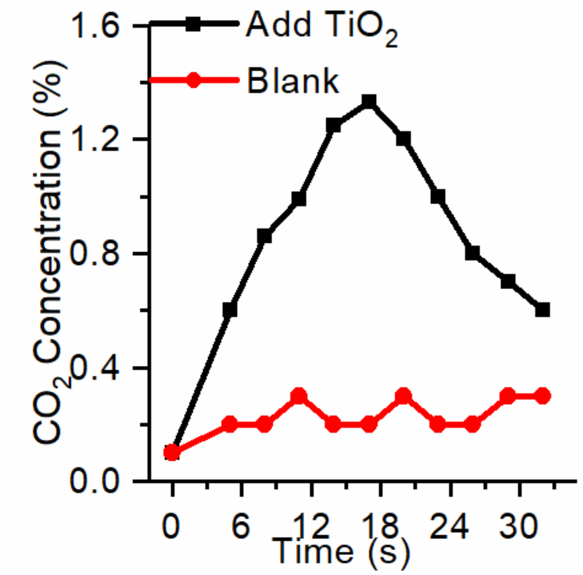

Figure S23. The recorded releasing of CO_2 upon adding TiO_2 to the solution of $H_2[Pd_2(\mu\text{-}CO)_2Cl_4]$. The control experiment was recorded before TiO_2 adding.

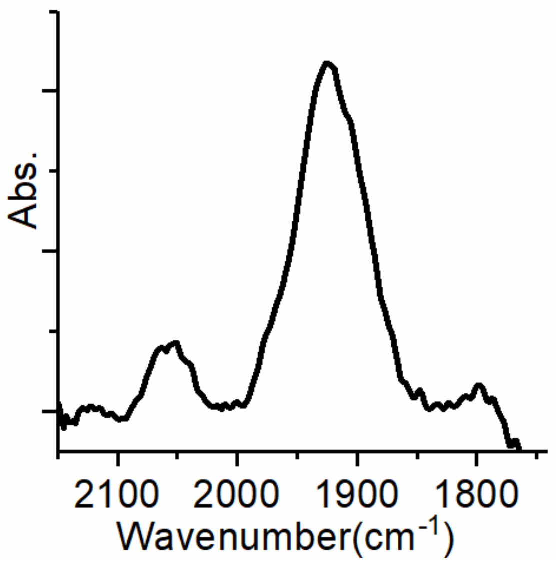

Figure S24. DRIFTS of the as-obtained Pd_2CO/TiO_2. The predominate bridge-site CO at ~ 1920 cm^{-1} was consistent with that in the precursor.[5]

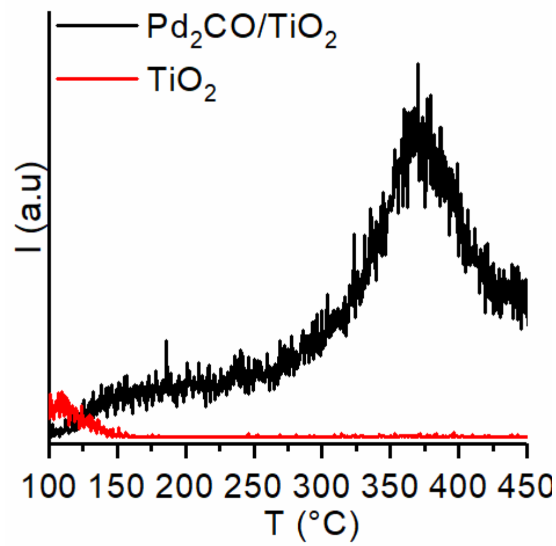

Figure S25. TPD-MS signal of CO recorded for blank TiO_2 and the as-obtained Pd_2CO/TiO_2. The desorption of CO over 300 °C implied that the adsorption of inherent CO in Pd_2CO/TiO_2 was very strong.

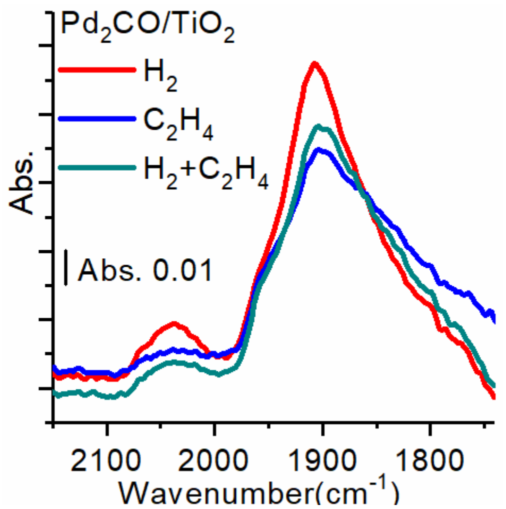

Figure S26. *In-situ* DRIFTS for Pd_2CO/TiO_2 in the condition of catalytic ethylene hydrogenation. It showed that the adsorbed CO molecules were preserved during ethylene hydrogenation.

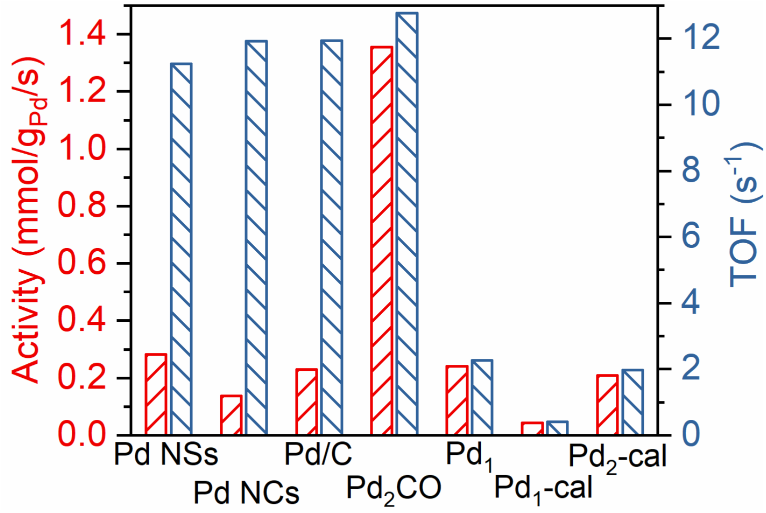

Figure S27. The calculated mass-specific activity and TOF of Pd nanosheets (Pd NSs), Pd nanocubes (Pd NCs), commercial Pd/C, Pd_1/TiO_2-EG (Pd_1), Pd_2CO/TiO_2 (Pd_2CO), Pd_1/TiO_2-cal (Pd_1-cal) and Pd_2/TiO_2-cal (Pd_2-cal) for the styrene hydrogenation carried out at 30 °C and 0.1 MPa H_2. The Pd dispersion was measured by CO titration and calculated with a Pd/CO ratio of 2. The Pd dispersions of Pd NSs, Pd NCs, and Pd/C were 23.7%, 10.8% and 18.1%. The Pd dispersions of Pd_2CO, Pd_1-cal and Pd_2-cal were hard to be measured due to the low mass loading, the dispersions of Pd on these catalysts were estimated to be close to 1. The calculated activity and TOF demonstrated that the supported Pd_2CO clusters were as active as the surface Pd in large NPs. More importantly, the high Pd dispersion made the supported Pd_2CO exhibit 4-6 times higher mass-specific activity.

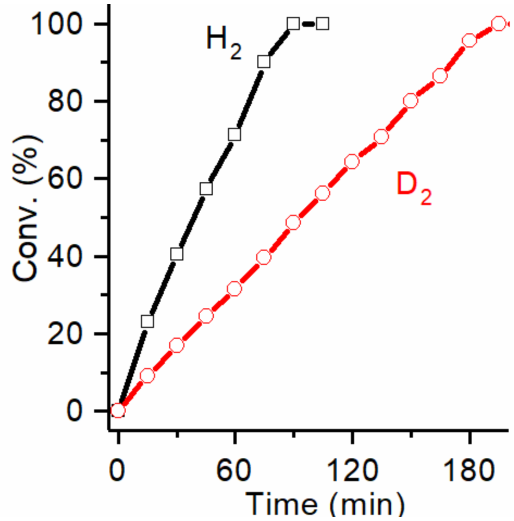

Figure S28. Kinetic isotopic experiment for Pd_2CO/TiO_2. The hydrogenation of styrene was carried out in H_2 or D_2 atmosphere. The k_H/k_D was calculated to be 2.02, which was much lower than that of the Pd_1/TiO_2-EG (k_H/k_D = 5.75).[2]

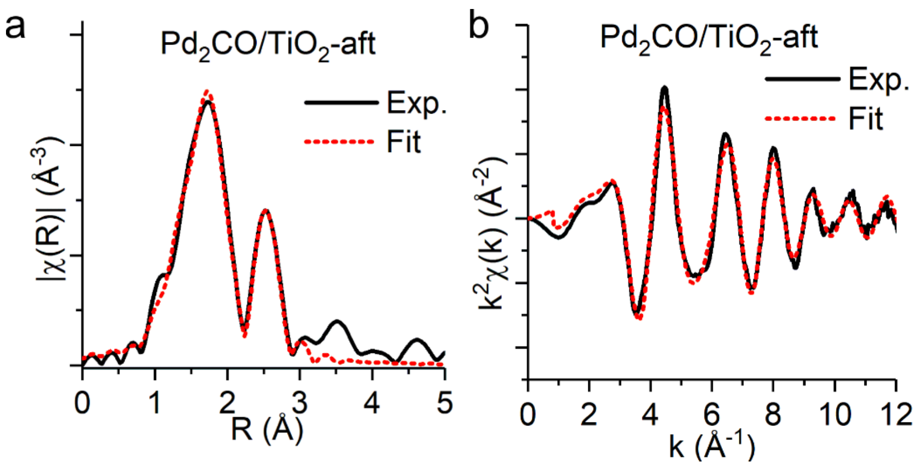

Figure S29. EXAFS (a) R-space and (b) k-space fitting of Pd_2CO/TiO_2 after styrene hydrogenation.

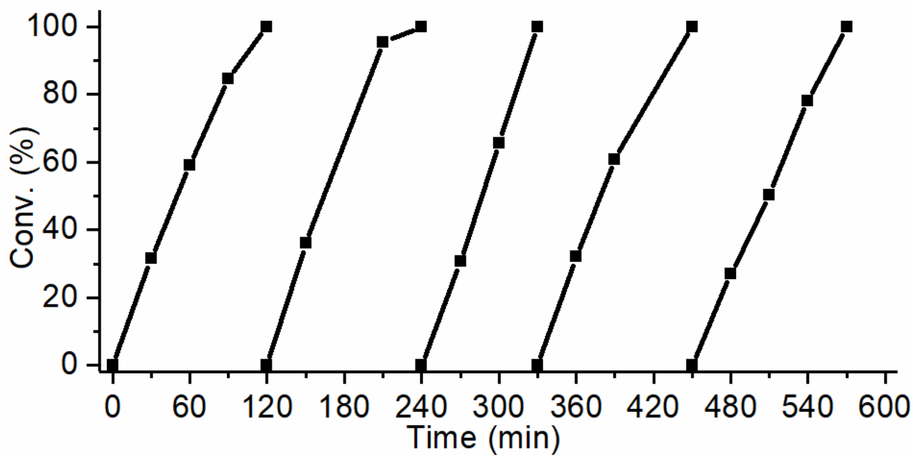

Figure S30. Catalytic performance of Pd_2CO/TiO_2 in five test rounds. Catalysis condition: 0.1 MPa H_2, 30 °C, S/C = 50,000, 10 mL EtOH, and 0.55 mL styrene (5 mmol) was introduced in every test round.

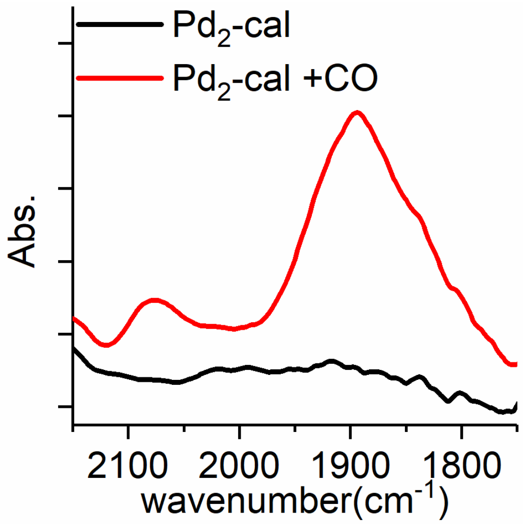

Figure S31. DRIFTS of Pd_2/TiO_2-cal before and after treated with CO.

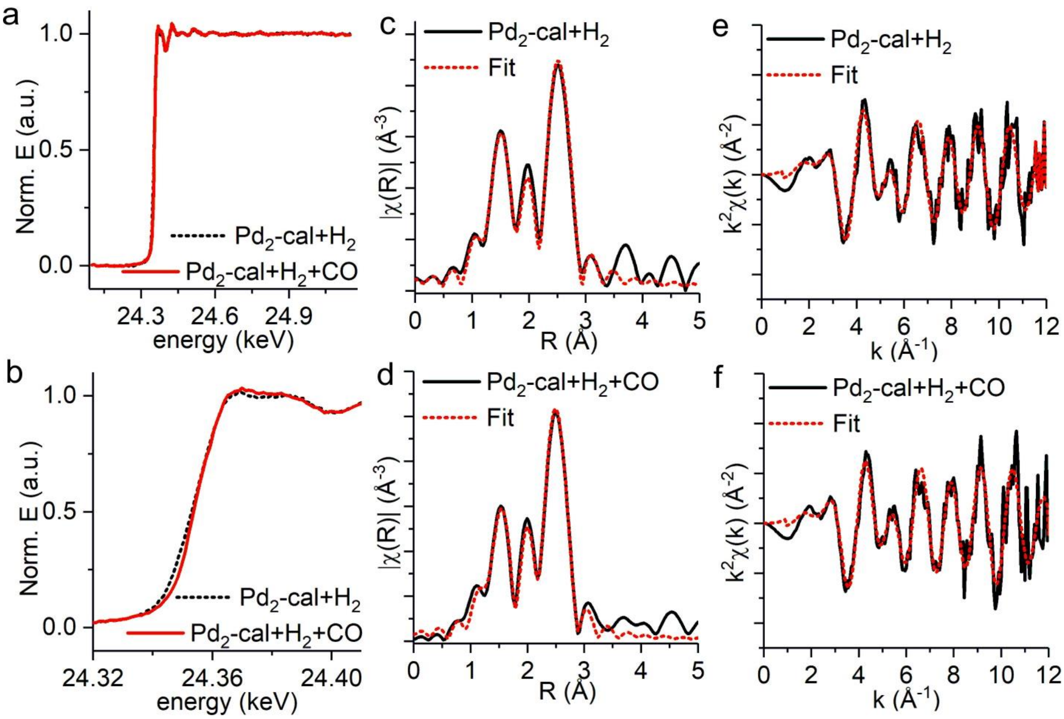

Figure S32. XAS (a), XANES, (b) EXAFS R-space (c, d) and k-space (e, f) fitting of Pd_2/TiO_2-cal after treated with H_2, and further with CO.

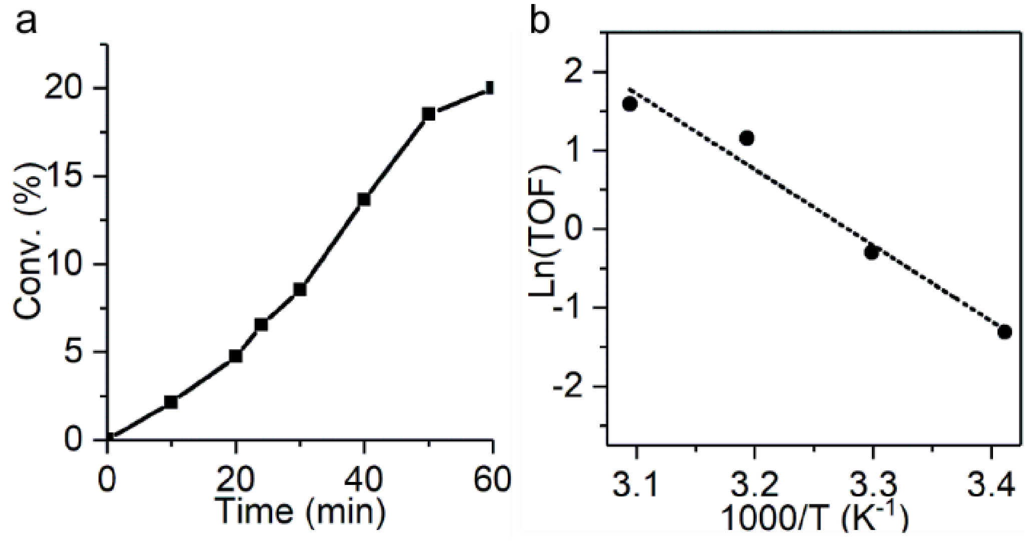

Figure S33. (a) Catalytic styrene hydrogenation performance of Pd_2/TiO_2-cal (S/C=50,000) 30 °C, 0.1 MPa H_2. (b) The Arrhenius plot for Pd_2/TiO_2-cal catalyzed styrene hydrogenation at 20, 30, 40 and 50 °C.

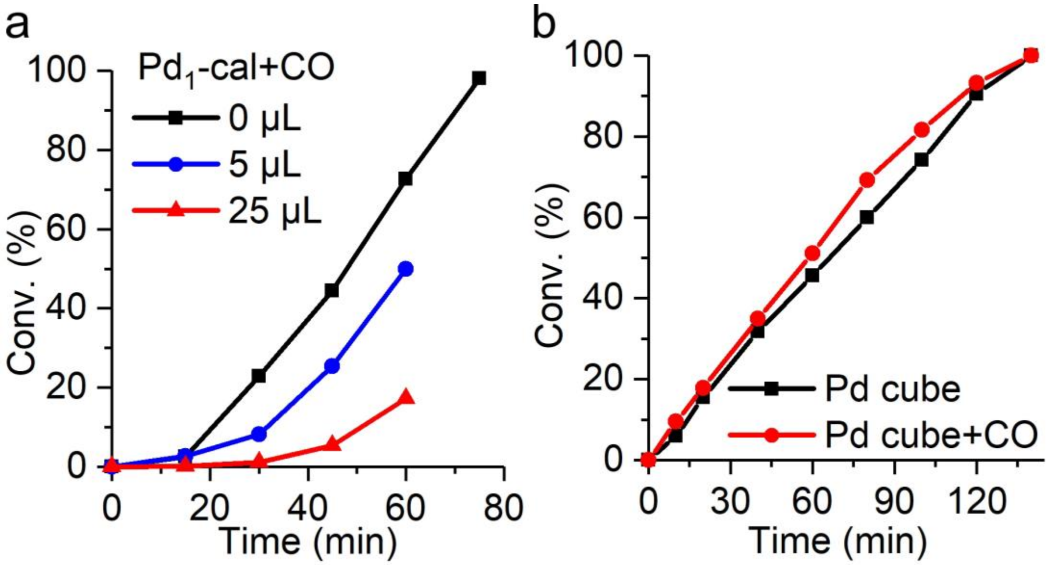

Figure S34. (a) Catalytic styrene hydrogenation performance of Pd_1/TiO_2-cal (S/C=2,000) with different amount of CO introduced. (b) Pd Cube (S/C = 10,000) with or without CO pre-treatment. Condition: 30 °C, 0.1 MPa H_2.

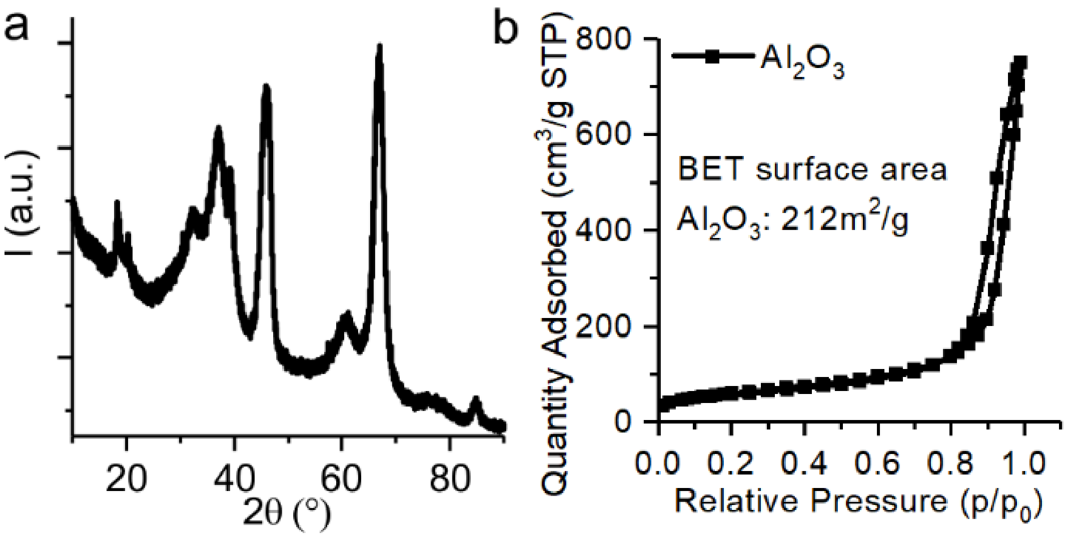

Figure S35. (a) The XRD pattern and (b) N_2 adsorption-desorption isotherm profile of γ-Al_2O_3.

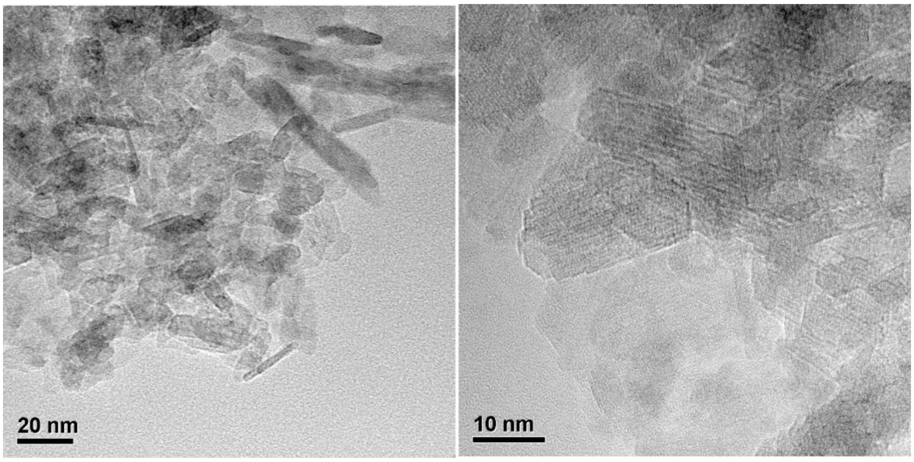

Figure S36. TEM images of 0.5 wt% Pd/Al_2O_3. No obvious Pd NP was figured out.

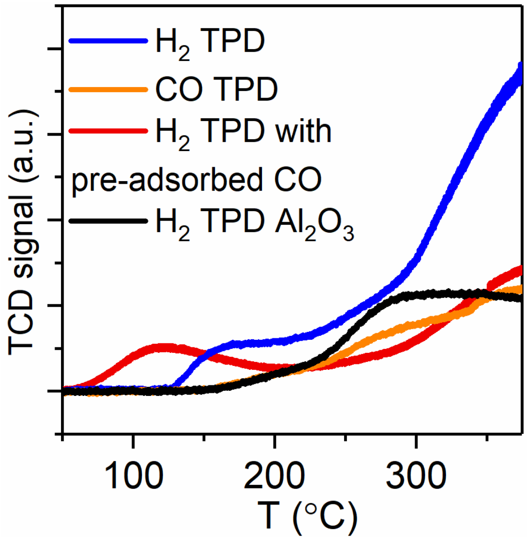

Figure S37. H_2 TPD (blue), CO TPD (yellow), and H_2 TPD with pre-adsorbed CO (red) over 0.5 wt% Pd/Al_2O_3. The H_2 TPD for blank Al_2O_3 (black) was also shown for comparison. The shoulder peak with desorption temperature of 175 °C in H_2 TPD was the desorption of H_2 from Pd, the higher-temperature desorption should be related to the dehydration of the support. For the CO TPD, despite the desorption of CO was hard to discriminate from the background, no desorption feature was observed below 150 °C. Similar to the following result of DRIFTS (almost no change was observed during temperature ramp from 60 °C to 100 °C), the CO TPD indicated the strong binding of CO on Pd cluster. More importantly, the H_2 desorption temperature decreased to about 120 °C when the sample was treated with CO first before H_2-TPD, which unambigously revealed that the coordination of CO reduced the binding energy of H_2 over small Pd clsuters.

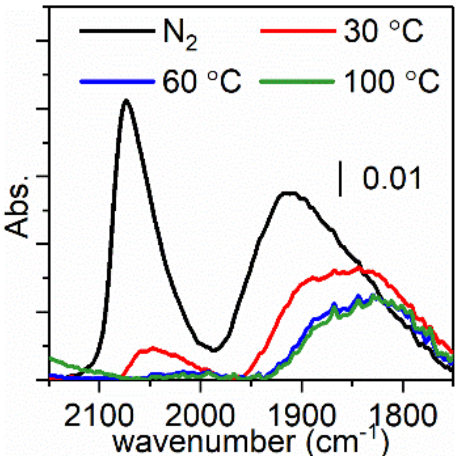

Figure S38. The *in-situ* DRIFTS for 0.5 wt% Pd/Al_2O_3 with pre-adsorbed CO in the catalytic ethylene hydrogenation condition at different temperature. The presence of bridge and hollow site adsorbed CO (1750 - 1950 cm^{-1}) suggested the formation of Pd-Pd bond after the pre-treatment.

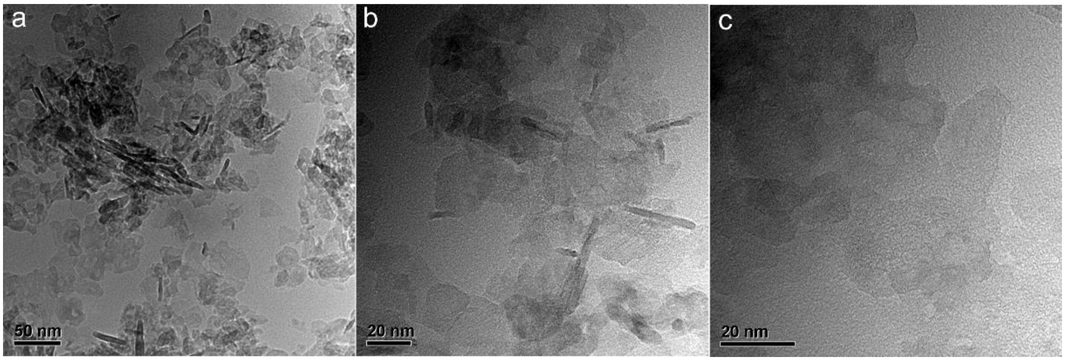

Figure S39. TEM images of 0.2 wt% Pd_2CO/Al_2O_3.

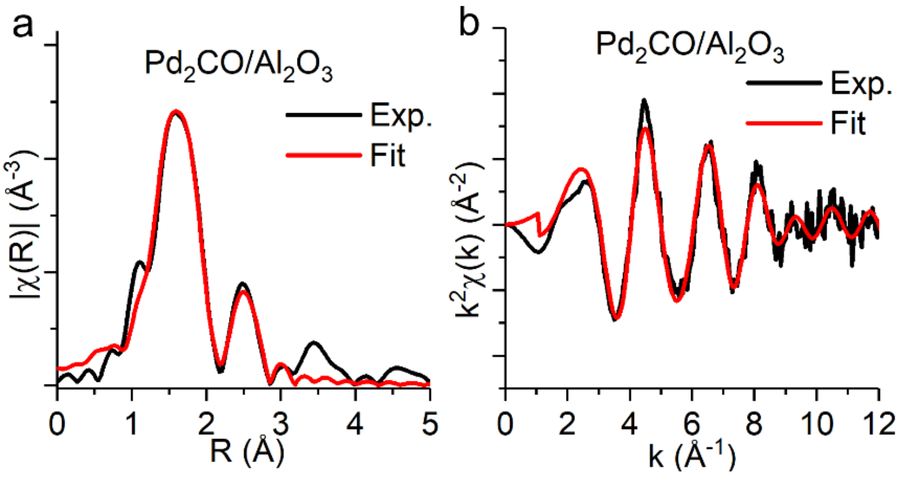

Figure S40. EXAFS (a) R-space and (b) k-space fitting of Pd_2CO/Al_2O_3.

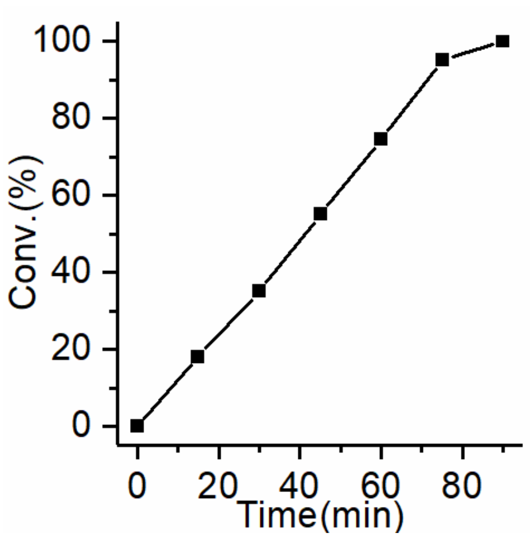

Figure S41. Styrene hydrogenation catalyzed by 0.2 wt% Pd_2CO/Al_2O_3.

Table S1. Adsorption energies of 2H (ΔE_{2H}, eV) for Pd_n (n=2, 3, 4, 7) clusters on TiO_2(010) surface in the absence or presence of CO.

**Structure**	**ΔE_{2H} (eV)**	
	**CO free**	**CO preadsorption**
Pd_2/TiO_2	-2.80	-0.17
Pd_3/TiO_2	-1.95	-0.58
Pd_4/TiO_2	-1.79	-1.21
Pd_7/TiO_2	-1.41	-1.08

Table S2. The Pd-Pd distances (unit: Å) of Pd_n (n=2, 3, 4, 7) and Pd_nCO clusters on TiO_2(010) surface (M/TiO_2) and the bare ones (M).

System	Pd_2	Pd_2CO	Pd_3	Pd_3CO	Pd_4	Pd_4CO	Pd_7	Pd_7CO
M	2.481	2.610	2.523<br>2.523<br>2.523	2.678<br>2.676<br>2.677	2.609<br>2.609<br>2.609<br>2.609<br>2.602<br>2.600	2.754<br>2.665<br>2.768<br>2.669<br>2.665<br>2.758	2.695<br>2.650<br>2.695<br>2.695<br>2.650<br>2.695<br>2.695<br>2.695<br>2.695<br>2.650<br>2.695<br>2.650<br>2.695<br>2.651<br>2.695	2.740<br>2.663<br>2.684<br>2.633<br>2.744<br>2.682<br>2.756<br>2.757<br>2.759<br>2.740<br>2.744<br>2.633<br>2.744<br>2.745
M/TiO_2	2.556	2.725	2.474<br>2.501<br>2.580	2.638<br>2.782<br>2.782	2.532<br>2.518<br>2.512<br>2.515	2.627 2.648 2.689 2.753	2.600<br>2.639<br>2.740<br>2.641<br>2.638<br>2.622<br>2.956<br>2.780<br>2.696<br>2.673<br>2.628<br>2.846<br>2.679<br>2.726<br>2.695	2.649<br>2.660<br>2.697<br>2.700<br>2.702<br>2.710<br>2.720<br>2.737<br>2.747<br>2.756<br>2.770<br>2.783<br>2.799<br>2.821<br>2.833

Table S3. The total magnetizations (unit: a.u.) of Pd in Pd_n (n=2, 3, 4, 7) and Pd_nCO clusters on TiO_2(010) surface (M/TiO_2) and the bare ones (M).

Magnetization	Pd_2	Pd_2CO	Pd_3	Pd_3CO	Pd_4	Pd_4CO	Pd_7	Pd_7CO
M	2.00	0.12	2.00	0.07	2.00	-0.14	2.00	0.02
M/TiO_2	1.96	0.07	-0.06	0.00	0.00	0.22	3.19	0.00

Table S4. The average bader charges (unit: a.u.) of Pd in Pd_n (n=2, 3, 4, 7) and Pd_nCO clusters on TiO_2(010) surface (M/TiO_2).

System	Average Bader charge	
	CO free	CO preadsorption
Pd_2/TiO_2	-0.06	0.15
Pd_3/TiO_2	0.05	0.14
Pd_4/TiO_2	0.04	0.11
Pd_7/TiO_2	0.06	0.09

Table S5. Crystal data and structure refinement for $(Ph_4P)_2[Pd_2(\mu\text{-}CO)_2Cl_4]$.

Empirical formula	$C_{25}H_{20}Cl_2OP$ Pd	
Formula weight	544.68	
Temperature	100 K	
Wavelength	1.54184 Å	
Crystal system, space group	Triclinic, P -1	
Unit cell dimensions	a = 9.4475(7) Å	alpha = 67.334(6)º
	b = 10.2295(8)Å	beta = 80.486(6)º
	c = 13.1863(8) Å	gamma = 71.144(7)º
Volume	1111.62(15) $Å^3$	
Z, Calculated density	2, 1.627 g/cm^3	
Absorption coefficient	9.738 mm^{-1}	
F(000)	546	
Crystal size	0.30x0.10x0.10mm^3	
Theta range for data collection	3.636 to 65.563 deg.	
Limiting indices	-11<=h<=11, -12<=k<=11, -14<=l<=15	
Reflections collected / unique	3633 / 3729 [$R_{(int)}$ = 0.0240]	
Completeness to theta = 61.08	99.85 %	
Refinement method	Full-matrix least-squares on F^2	
Data / restraints / parameters	3729 /0 / 271	
Goodness-of-fit on F^2	1.059	
Final R indices [I>2sigma(I)]	R_1 = 0.0266, wR_2 = 0.0726	
R indices (all data)	R_1 = 0.0271, wR_2 = 0.0730	
Largest diff. peak and hole	0.615 and -0.791 e. $Å^{-3}$	

Table S6. Atomic coordinates (×10^4) and equivalent isotropic displacement parameters (Å^{2}x10^3) for $(Ph_4P)_2[Pd_2(\mu\text{-}CO)_2Cl_4]$: U(eq) was defined as one-third of the trace of the orthogonalized U_{ij} tensor.

	**x**	**y**	**z**	**U(eq)**
Pd1	0.07772(2)	0.97379(2)	0.41215(2)	0.01279(9)
Cl1	0.03978(7)	1.08967(7)	0.22060(5)	0.02006(15)
Cl2	0.29993(7)	0.79577(7)	0.38537(5)	0.01989(15)
P1	0.62454(7)	0.59105(7)	0.74186(5)	0.01223(14)
O1	0.2264(2)	0.8209(2)	0.62233(16)	0.0309(5)
C25	0.4328(3)	0.5202(3)	0.6443(2)	0.0156(5)
C14	0.7722(3)	0.4504(3)	0.8261(2)	0.0139(5)
C7	0.6243(3)	0.8237(3)	0.7978(2)	0.0167(5)
C9	0.6031(3)	0.7904(3)	0.5289(2)	0.0168(5)
C20	0.4899(3)	0.5026(3)	0.7410(2)	0.0136(5)
C17	0.9998(3)	0.2248(3)	0.9501(2)	0.0172(5)
C19	0.8288(3)	0.3177(3)	0.8060(2)	0.0166(5)
C15	0.8293(3)	0.4693(3)	0.9083(2)	0.0161(5)
C2	0.5382(3)	0.7345(3)	0.7988(2)	0.0147(5)
C18	0.9441(3)	0.2057(3)	0.8680(2)	0.0171(5)
C10	0.6592(3)	0.8595(3)	0.4251(2)	0.0195(6)
C24	0.3229(3)	0.4539(3)	0.6504(2)	0.0188(6)
C5	0.4190(3)	0.9519(3)	0.8900(2)	0.0200(6)
C22	0.3283(3)	0.3534(3)	0.8477(2)	0.0207(6)
C13	0.8531(3)	0.6321(3)	0.5798(2)	0.0160(5)
C4	0.3336(3)	0.8660(3)	0.8882(2)	0.0196(6)
C11	0.8121(3)	0.8172(3)	0.3988(2)	0.0186(6)
C8	0.6996(3)	0.6757(3)	0.6063(2)	0.0148(5)
C16	0.9425(3)	0.3559(3)	0.9710(2)	0.0183(5)
C6	0.5638(3)	0.9324(3)	0.8437(2)	0.0199(6)
C3	0.3923(3)	0.7562(3)	0.8425(2)	0.0161(5)
C21	0.4389(3)	0.4171(3)	0.8432(2)	0.0179(5)
C23	0.2700(3)	0.3721(3)	0.7515(2)	0.0207(6)
C12	0.9080(3)	0.7038(3)	0.4757(2)	0.0194(6)
C1	0.1256(3)	0.8933(3)	0.5692(2)	0.0207(6)

Table S7. EXAFS fitting results.

Sample	Shell	C.N.	R (Å)	$\sigma^2\times10^3$(Å^2)	E_0 shift (eV)	R-factor
Pd foil	Pd-Pd	12*	2.74±0.02	5.4±0.2	-5.1±0.3	0.003
Pd_2CO cluster	Pd-(CO)	2.0±0.8	1.96±0.04	7.1±6.0	-4.8±6.1	0.008
	Pd-Cl	2.0±0.7	2.38±0.01	6.1±2.5	4.0±1.8	
	Pd-Pd	1.1±0.6	2.69±0.02	3.7±3.3	7.6±4.4	
Pd_2CO/TiO_2	Pd-O	2.4±0.6	2.03±0.04	4.8±3.7	6.5±3.8	0.007
	Pd-Pd	1.2±0.4	2.76±0.03	5.8±5.5	3.7±5.6	
Pd_2CO/TiO_2-aft	Pd-O	1.8±0.5	2.02±0.07	7.2±5.4	4.6±6.1	0.005
	Pd-Pd	2.8±1.0	2.76±0.02	7.3±2.6	4.3±2.8	
Pd_2/TiO_2-cal+H_2	Pd-O	1.2±0.2	2.01±0.02	2.1±1.1	3.0±2.1	0.005
	Pd-Pd	4.3±0.5	2.76±0.01	7.1±0.9	-4.2±0.8	
Pd_2/TiO_2-cal+H_2+CO	Pd-O	1.1±0.2	1.99±0.01	1.9±0.8	3.1±2.0	0.005
	Pd-Pd	4.3±0.4	2.76±0.01	6.5±0.7	-4.4±0.7	
Pd_2CO/Al_2O_3	Pd-O	2.6±0.7	2.02±0.03	3.7±3.2	5.8±2.8	0.005
	Pd-Pd	1.3±1.0	2.77±0.03	6.4±5.2	4.9±4.8	

Table S8. Production efficiency of the H_2O_2 following the 2-eAQ hydrogenation route catalyzed by different Pd catalysts reported.

Cat.	Pd loading (wt%)	Temp. (℃)	H_2 Press. (bar)	Yield (%)	Rate (g_{H2O2}/g_{Pd}/h)	Ref.
Pd/Al_2O_3	1.0	20	\	93	63.24	[23]
Pd/Al_2O_3	0.50	40	\	96.4	32.9	[24]
Pd/SiO_2	1.0	50	6.8	50	1280	[25]
Pd/Al_2O_3	0.2	60	1.0	38.2	442.7	[26]
$PdIr/Al_2O_3$	0.75	50	3.0	92.1	2050	[27]
Pd/Al_2O_3	1.0	50	3.0	61	1110	[27]
Pd/glass	2.40	50	3.0	60	3800	[28]
Pd/glass	0.32	70	3.5	35.5	567.5	[29]
Pd/Al_2O_3	1.0	80	3.0	95.7	3610	[30]
Pd_2CO/Al_2O_3	0.20	30	2	93	1054	This work

REFERENCES

[1] G. Xiang, T. Li, J. Zhuang, X. Wang, "Large-scale synthesis of metastable TiO_2(B) nanosheets with atomic thickness and their photocatalytic properties." Chem. Commun., vol. 46, no. 36, pp. 6801-6803, 2010.
[2] P. Liu, Y. Zhao, R. Qin *et al.*, "Photochemical route for synthesizing atomically dispersed palladium catalysts." Science, vol. 352, no. 6287, pp. 797-801, 2016.
[3] P. Liu, Y. Zhao, R. Qin *et al.*, "A vicinal effect for promoting catalysis of Pd_1/TiO_2 : supports of atomically dispersed catalysts play more roles than simply serving as ligands." Sci. Bull., vol. 63, no. 11, pp. 675-682, 2018.
[4] X. J. Zhao, Y. Zhao, G. Fu, N. F. Zheng, "Origin of the facet dependence in the hydrogenation catalysis of olefins: experiment and theory." Chem. Commun., vol. 51, no. 60, pp. 12016-12019, 2015.
[5] H. Li, G. X. Chen, H. Y. Yang *et al.*, "Shape-Controlled Synthesis of Surface-Clean Ultrathin Palladium Nanosheets by Simply Mixing a Dinuclear Pd^I Carbonyl Chloride Complex with H_2O." Angew. Chem. Int. Ed., vol. 52, no. 32, pp. 8368-8372, 2013.
[6] M. D. Rossell, F. J. Caparrós, I. Angurell *et al.*, "Magnetite-supported palladium single-atoms do not catalyse the hydrogenation of alkenes but small clusters do." Catal. Sci. Technol., vol. 6, no. 12, pp. 4081-4085, 2016.
[7] B. Ravel, M. Newville, "ATHENA, ARTEMIS, HEPHAESTUS: data analysis for X-ray absorption spectroscopy using IFEFFIT." J. Synchrotron Radiat., vol. 12, no. 4, pp. 537-541, 2005.
[8] G. Kresse, J. Hafner, "Ab initio molecular dynamics for open-shell transition metals." Phys. Rev. B, vol. 48, no. 17, pp. 13115-13118, 1993.
[9] G. Kresse, J. Furthmüller, "Efficient iterative schemes for ab initio total-energy calculations using a plane-wave basis set." Phys. Rev. B, vol. 54, no. 16, pp. 11169-11186, 1996.
[10] J. P. Perdew, K. Burke, M. Ernzerhof, "Generalized Gradient Approximation Made Simple." Phys. Rev. Lett., vol. 78, no. 7, pp. 1396-1396, 1997.
[11] G. Kresse, D. Joubert, "From ultrasoft pseudopotentials to the projector augmented-wave method." Phys. Rev. B, vol. 59, no. 3, pp. 1758-1775, 1999.
[12] P. E. Blöchl, "Projector augmented-wave method." Phys. Rev. B, vol. 50, no. 24, pp. 17953-17979, 1994.
[13] G. Henkelman, B. P. Uberuaga, H. Jónsson, "A climbing image nudged elastic band method for finding saddle points and minimum energy paths." Journal of Chemical Physics, vol. 113, no. 22, pp. 9901-9904, 2000.
[14] H.-W. Lee, C.-M. Chang, "Size effect of Pd clusters on hydrogen adsorption." J. Phys.: Condens. Matter, vol. 23, no. 4, pp. 045503, 2011.
[15] V. Kumar, Y. Kawazoe, "Icosahedral growth, magnetic behavior, and adsorbate-induced metal-nonmetal transition in palladium clusters." Physical Review B, vol. 66, no. 14, pp. 144413, 2002.
[16] B. J. Morgan, G. W. Watson, "A DFT+U description of oxygen vacancies at the TiO2 rutile (110) surface." Surface Science, vol. 601, no. 21, pp. 5034-5041, 2007.
[17] N. M. Dimitrijevic, Z. V. Saponjic, B. M. Rabatic, O. G. Poluektov, T. Rajh, "Effect of Size and Shape of Nanocrystalline TiO_2 on Photogenerated Charges. An EPR Study." J. Phys. Chem. C, vol. 111, no. 40, pp. 14597-14601, 2007.

[18] R. F. Howe, M. Gratzel, "EPR observation of trapped electrons in colloidal titanium dioxide." J. Phys. Chem., vol. 89, no. 21, pp. 4495-4499, 1985.
[19] P. D. Harvey, Z. Murtaza, "Properties of PdI-PdI bonds. Theoretical and spectroscopic study of palladium $Pd_2(dmb)_2X_2$ complexes (dmb = 1,8-diisocyano-p-menthane; X = Cl, Br)." Inorg. Chem., vol. 32, no. 22, pp. 4721-4729, 1993.
[20] N. M. Kostic, R. F. Fenske, "Molecular orbital study of dinuclear palladium carbonyl chlorides. Choice of the bridging ligand (carbonyl vs. chloro) and the question of metal-metal bonding." Inorg. Chem., vol. 22, no. 4, pp. 666-671, 1983.
[21] P. D. Harvey, K. Hierso, P. Braunstein, X. Morise, "Comparison in halide binding ability between the unsaturated clusters $[Pd_3(\mu\text{-dppm})_3(CO)]^{2+}$ and $[PdPtCo(\mu\text{-dppm})_2(CO)_3(CNtBu)]^+$ (dppm = $Ph_2PCH_2PPh_2$)." Inorg. Chim. Acta, vol. 250, no. 1, pp. 337-343, 1996.
[22] H. Li, G. Chen, H. Yang *et al.*, "Shape-Controlled Synthesis of Surface-Clean Ultrathin Palladium Nanosheets by Simply Mixing a Dinuclear Pd^I Carbonyl Chloride Complex with H_2O." Angew. Chem. Int. Ed., vol. 52, no. 32, pp. 8368-8372, 2013.
[23] A. Biffis, R. Ricoveri, S. Campestrini *et al.*, "Highly Chemoselective Hydrogenation of 2-Ethylanthraquinone to 2-Ethylanthrahydroquinone Catalyzed by Palladium Metal Dispersed inside Highly Lipophilic Functional Resins." Chem. Eur. J., vol. 8, no. 13, pp. 2962-2967, 2002.
[24] H. Chen, D. Huang, X. Su *et al.*, "Fabrication of $Pd/\gamma\text{-}Al_2O_3$ catalysts for hydrogenation of 2-ethyl-9,10-anthraquinone assisted by plant-mediated strategy." Chem. Eng. J., vol. 262, no. pp. 356-363, 2015.
[25] R. Halder, A. Lawal, "Experimental studies on hydrogenation of anthraquinone derivative in a microreactor." Catal. Today, vol. 125, no. 1, pp. 48-55, 2007.
[26] Q. Wang, L. Wang, Y. Wang *et al.*, "Study on deactivation and regeneration of Pd/Al_2O_3 catalyst in hydrogen peroxide production by the anthraquinone process." React. Kinet. Catal. Lett., vol. 81, no. 2, pp. 297-304, 2004.
[27] R. Hong, Y. He, J. Feng, D. Li, "Fabrication of supported $Pd\text{-}Ir/Al_2O_3$ bimetallic catalysts for 2-ethylanthraquinone hydrogenation." Aiche J., vol. 63, no. 9, pp. 3955-3965, 2017.
[28] C. Shen, Y. J. Wang, J. H. Xu, Y. C. Lu, G. S. Luo, "Preparation and the hydrogenation performance of a novel catalyst-Pd nanoparticles loaded on glass beads with an egg–shell structure." Chem. Eng. J., vol. 173, no. 1, pp. 226-232, 2011.
[29] H. Yao, C. Shen, Y. Wang, G. Luo, "Catalytic hydrogenation of 2-ethylanthraquinone using an in situ synthesized Pd catalyst." RSC Adv., vol. 6, no. 28, pp. 23942-23948, 2016.
[30] E. Yuan, C. Wu, G. Liu, L. Wang, "One-pot synthesis of Pd nanoparticles on ordered mesoporous Al_2O_3 for catalytic hydrogenation of 2-ethyl-anthraquinone." Appl. Catal. A, vol. 525, no. pp. 119-127, 2016.
